# Reproductive Failure in UK Harbour Porpoises *Phocoena phocoena*: Legacy of Pollutant Exposure?

**DOI:** 10.1371/journal.pone.0131085

**Published:** 2015-07-22

**Authors:** Sinéad Murphy, Jonathan L Barber, Jennifer A. Learmonth, Fiona L. Read, Robert Deaville, Matthew W. Perkins, Andrew Brownlow, Nick Davison, Rod Penrose, Graham J. Pierce, Robin J. Law, Paul D. Jepson

**Affiliations:** 1 Institute of Zoology, Zoological Society of London, Regent’s Park, London, United Kingdom; 2 Centre for Environment, Fisheries and Aquaculture Science, Lowestoft, United Kingdom; 3 Oceanlab, University of Aberdeen, Newburgh, Aberdeenshire, United Kingdom; 4 Scottish Marine Animal Stranding Scheme, SRUC Veterinary Services Drummondhill, Inverness, United Kingdom; 5 Marine Environmental Monitoring, Penwalk, Llechryd, Cardigan, Ceredigion, United Kingdom; The University of Iowa, UNITED STATES

## Abstract

Reproductive failure in mammals due to exposure to polychlorinated biphenyls (PCBs) can occur either through endocrine disrupting effects or via immunosuppression and increased disease risk. To investigate further, full necropsies and determination of summed 25 polychlorinated biphenyls congeners (∑PCBs lipid weight) in blubber were undertaken on 329 UK-stranded female harbour porpoises (1990-2012). In sexually mature females, 25/127 (19.7%) showed direct evidence of reproductive failure (foetal death, aborting, dystocia or stillbirth). A further 21/127 (16.5%) had infections of the reproductive tract or tumours of reproductive tract tissues that could contribute to reproductive failure. Resting mature females (non-lactating or non-pregnant) had significantly higher mean ∑PCBs (18.5 mg/kg) than both lactating (7.5 mg/kg) and pregnant females (6 mg/kg), though not significantly different to sexually immature females (14.0 mg/kg). Using multinomial logistic regression models ΣPCBs was found to be a significant predictor of mature female reproductive status, adjusting for the effects of confounding variables. Resting females were more likely to have a higher PCB burden. Health status (proxied by “trauma” or “infectious disease” causes of death) was also a significant predictor, with lactating females (i.e. who successfully reproduced) more likely to be in good health status compared to other individuals. Based on contaminant profiles (>11 mg/kg lipid), at least 29/60 (48%) of resting females had not offloaded their pollutant burden via gestation and primarily lactation. Where data were available, these non-offloading females were previously gravid, which suggests foetal or newborn mortality. Furthermore, a lower pregnancy rate of 50% was estimated for “healthy” females that died of traumatic causes of death, compared to other populations. Whether or not PCBs are part of an underlying mechanism, we used individual PCB burdens to show further evidence of reproductive failure in the North-east Atlantic harbour porpoise population, results that should inform conservation management.

## Introduction

Reproductive failure in marine mammals has been associated with exposure to persistent organic pollutants (POP), the effects of which were reported to occur at many stages of the reproductive cycle including implantation failure [1] and foetal death (abortion) in seals [2], and increased first-born calf mortality in bottlenose dolphins (*Tursiops truncatus*) [3]. Additionally, sterility caused by stenosis, occlusions and leiomyomas were reported in Baltic seals [2, 4, 5], the presence of luteinised cysts with the potential to impede ovulation were observed on the ovaries of Mediterranean striped dolphins (*Stenella coeruleoalba*) [6], and severe reproductive dysfunction through the development of cancer and hermaphroditism was documented in St Lawrence Estuary beluga whales (*Delphinapterus leucas*) [7–9]; again all associated with high levels of exposure to POPs, specifically organochlorines (OCs) such as polychlorinated biphenyls (PCBs) and dichlorodiphenyltrichloroethane (DDT). However, in some cases there have been difficulties in linking these proposed toxicological endpoints of reproductive dysfunction to high POP burdens due to the presence of confounding factors. For example, it is not known if the presence of ovarian luteinised cysts and lower fecundity, manifested in a high number of abortions, in Mediterranean striped dolphins that died during a morbillivirus epizootic were caused by high PCB levels, the morbillivirus infection or a combination of both [6]. In the early seventies, stillbirths and premature pupping in California sea lions (*Zalophus californianus californianus*) were attributed to the contamination by tDDT (total DDT) [10]. This relationship though was confounded by the presence of an enzootic bacterial disease (leptospirosis) capable of inducing abortions and, in other cases, prematurity was associated with nutritional stress caused by environmental variability [11]. While the levels of tDDT in California sea lions declined by one order of magnitude from 1970 to 2000 [12], marine biotoxins such as domoic acid, a naturally occurring algal toxin, has more recently been linked to (contributing to) reproductive failure in sea lions in this region [13].

The effects of PCBs and DDT on reproduction in the genetically distinct North-east Atlantic continuous system harbour porpoise (*Phocoena phocoena*) population, that ranges from France to Norway (and found primarily on continental shelf waters) including the North Sea [14], is a particular concern. Although the use and production of PCBs in Europe was phased out in the 1980s, diffuse inputs into the marine environment continue and environmental levels in marine biota (fish and mussels) are either declining slowly, or there is no general improvement [15]. Harbour porpoises, like other marine mammals feeding at higher trophic levels and with large lipid storage, will have increased risk of individual and population level toxicity from exposure to OCs due to limited capacity to metabolise and excrete these compounds [16]. Although an initial decline in blubber Σ25CBs concentrations was observed in UK porpoises sampled in the mid-1990s, this plateaued off after 1998 [17]. In contrast, levels of blubber ΣDDT significantly declined since the early 1990s, reflecting a lack of fresh inputs of this agricultural chemical [17].

Harbour porpoises are small cetaceans with high parasitic exposure, and individual health status often present energetic “knife-edge” conditions. General health status may be further exacerbated by adding additional health complications arising from high pollutant burdens. This may be reflected in the relationship between PCB exposure and infectious disease mortality in UK harbour porpoises, with significantly higher blubber PCB levels reported in individuals that died from infectious disease compared to physical trauma [18]. A low estimated pregnancy rate compared to other geographic areas was recently reported for harbour porpoises inhabiting Scottish waters (30–40%, sampling period 1990–2005 [19]; and for porpoises in western European waters (42%; sampling period 2001–2003; [20], which possibly suggests reproductive failure/impairment in the population. As the vast majority of individuals sampled within these studies however were stranded animals that died as a result of numerous pathological reasons, such as generalized bacterial infection, pneumonia and starvation, in addition to trauma, it is not known if they are really representative samples of the extant population. In order to investigate this further, the current study assesses pregnancy rates and evidence of reproductive failure in female harbour porpoises, using case (infectious disease) and control (trauma) cause of death groups, and investigates their association with exposure to PCBs and DDT. Confounding factors, such as age, nutritional status and exposure to infectious disease, lead to difficulties in specifically attributing the presence of higher OCs to reproductive disorders in previous studies on marine mammals [21]. These factors will be controlled for within the current study.

## Materials and Methods

### Gross post-mortem examinations

The UK Cetacean Stranding Investigation Programme conducts work on UK strandings under contract to the UK Department of Environment, Food and Rural Affairs. It has appropriate licenses from the relevant authorities (Natural England, Scottish Natural Heritage and Natural Resources Wales) to allow it to collect and hold carcasses and samples from European Protected Species, in line with UK legislation enacted under the EU Habitats Directive. No animals were killed or harmed for the purposes of this study. Gross necropsy and tissue sampling protocols followed European Cetacean Society guidelines [22]. Condition of individuals was fresh-to-moderate decomposition on necropsy, with the majority in fresh (68%) or slight (27%) decomposition. Basic data collected from each animal included stranding location, date, sex, total length, ventral blubber thickness (along the ventral abdomen in front of the dorsal fin) and girth (in front of the dorsal fin). Causes of death (COD) were determined by specific diagnostic criteria (see [18, 23], and individuals were categories in to three COD groups: infectious disease, trauma (bycatch, boat/ship strike, bottlenose dolphin attacks and dystocia) and others (live stranding, starvation, neoplasia and not established).

During gross examinations, the reproductive tracts of sexually mature individuals were examined for the presence of a foetus, evidence of female aborting a calf, dystocia and foetal death. Additionally in mature individuals, reproductive tracts were assessed for cases of sterility and infertility from stenosis, occlusions, leiomyoma and endometriosis, and other reproductive tract abnormalities. Where possible, occurrences of disease and infection were confirmed by bacteriology, virology and histopathology assessments. In order to assess previous gravidity, the development of the blood vessels in the broad ligaments and the presence of striations within the smooth musculature of the uterine wall were noted, as well as the degree of uterine involution. Mammary glands were examined for evidence of lactation via gross examination and, in some individuals, histological assessment of mammary tissue. Frozen blubber and teeth samples (-20°C) and 10% neutral buffered formalin fixed reproductive material were retained for further investigations.

### Analysis of chemical toxins

Blubber samples were analysed by the Centre for Environment, Fisheries, and Aquaculture Science Laboratory for quantification of hexane extractable lipid and wet weight concentrations (mg/kg) of 25 individual chlorobiphenyl (IUPAC numbers: 18, 28, 31, 44, 47, 49, 52, 66, 101, 105, 110, 118, 128, 138, 141, 149, 151, 153, 156, 158, 170, 180, 183, 187, 194) and a range of organochlorine pesticides (p,p’-DDE, p,p’-TDE (also known as p,p’-DDD) and p,p’-DDT) using previously established and validated protocols based on international standardised methodologies (see [17, 18]). Concentrations were converted to a lipid weight basis (mg/kg lipid) using the proportion of hexane extractable lipid in each individual blubber sample, and the sum of the concentrations of the 25 PCB congeners (ΣPCBs mg/kg lipid) and three DDT congeners (ΣDDT mg/kg lipid) were determined. For all analyses, appropriate quality control materials (certified or laboratory reference materials) were analysed within each sample batch in order that the day-to-day performance of the methods could be monitored [24].

### Determination of age and reproductive status

The straightest and least worn teeth were selected for age estimation from the middle section of the lower jaw. Age was determined by analysing growth layer groups (GLGs) in the dentine tissue of dental samples, following Lockyer [25]. Teeth were decalcified, sectioned and the most central and complete sections (including the whole pulp cavity) were selected from each tooth, stained, mounted on glass slides, and allowed to dry. GLGs were counted under a binocular microscope and on enhanced computer images of the sections. All readings were initially made blind (with no access to other data on the animals) and replicate counts were made by at least two readers. In cases where there was disagreement (>1 year disparity), teeth were re-examined by readers and an age and/or an age range was agreed. As ages were recorded by a number of different researchers, cross-calibration exercises were carried out.

Fixed ovarian samples were assessed externally for the presence of corpora scars (corpus luteum and albicans) and then hand-sectioned into 0.5–2 mm slices and examined internally under a binocular microscope for the presence of additional corpora scars. Females were considered sexually mature if their ovaries contained at least one corpus luteum or albicans. Total numbers of corpora scars (number of ovulations) were counted, which was used an index of reproductive activity (after Murphy et al. [26]).

### Statistical analyses

#### Estimation of reproductive parameters

Assessment of female reproductive status follows the procedures and terminology recommended by the International Whaling Commission [27]. Females were classified into five reproductive states: (1) sexually immature, (2) pregnant (foetus present), (3) pregnant and lactating, (4) sexually mature and lactating and (5) resting mature (not pregnant or lactating) [26]. The pregnancy rate (PR) was estimated by calculating the proportion of pregnant females in the sexually mature sample. Only females sampled outside the conception period for the species in UK waters (May-September; [19]) were included in the analysis, due to the increased possibility that embryos or small foetuses were not detected during early stages of gestation.

Average age at sexual maturity (ASM) and its variance was estimated using the sum of fraction of immature algorithm [28].

$$\text{ASM = }j+\overset{K}{\underset{i=j}{\sum }}p_{i}$$

$$\text{Variance }\left({\text{s}}^{2}\right)\text{= }\sum \frac{p_{i}q_{i}}{N_{i}-1}$$

Where, if I_i_ ≠ N_i_, p_i_ = I_i_ / N_i_, and q_i_ = (M_i_) /N_i;_ if I_i_ = N_i_, p_i_ = (I_i_ - ½)/N_i_, and q_i_ = (M_i_ + ½)/N_i_, and if M_i_ = N_i_, p_i_ = (I_i_ + ½)/N_i_, and q_i_ = (M_i_ - ½)/N_i_.

Confidence interval (at p = 0.05) = ASM ± 1.96 √s^2^

j = the first indeterminate age class

k = the last indeterminate age class

p_i_ = fraction of immature specimens in age class i

q_i_ = fraction of mature specimens in age class i (p_i_ + q_i_ = 1)

I_i_ = number of immature specimens in age class i

M_i_ = number of mature specimens in age class i

N_i_ = number of specimens in age class i (N_i_ = I_i_ + M_i_)

#### Association between PCBs and reproductive status, age and COD class

Primiparous and multiparous female cetaceans can offload their organochlorine pollutant burden through transplacental transfer and also via lactation as OCs are mobilised from blubber to lipid rich milk [3]. Individual studies reported that in bottlenose dolphins, the majority (c. 80% of OCs) of a female’s contaminant burden is believed to be transferred to first born calves during early lactation [29]. A similar estimation was reported for striped dolphins where 72–91% of total body burdens were offloaded during lactation, whereas only 4–9% of total body burdens wereoffloaded during gestation ([30] reported in [31]). Further, depending on age and female reproductive history, OC transfer estimates of 60–100% during lactation and 4–10% during gestation were reported in long-finned pilot whales (*Globicephala melas*) [32]. In order to assess offloading of pollutant burdens in mature female harbour porpoises, linear regression analysis was undertaken with ΣPCBs (mg/kg) as the dependent variable and corpora scar number and age (years) as independent variables. Pattern of offloading was compared between females in different reproductive status categories. Natural log transformations were undertaken prior to regression analysis so as to stabilise the variance.

Accumulation of PCBs in harbour porpoises was assessed using available data from male porpoises that stranded along UK coastlines between 1990 and 2012 (n = 263), as males do not offload their contaminant load during their lifetime. A linear model was applied to the relationship between blubber PCB concentrations (mg/kg) and age (years) (after Hall et al. [33]) and, because of large-scale individual variation in ΣPCBs at a given age, outliers more than three standard residuals from the fitted line were removed from the analysis.

General linear models were undertaken with Tukey’s post-hoc tests to compare mean (LN transformed) ΣPCBs mg/kg concentrations among female reproductive status categories and also COD groups.

Blubber “total PCBs” toxicity threshold concentration for the onset of physiological endpoints in marine mammals of 17mg/kg lipid (as Aroclor 1254) [34] was converted to the equivalent value of 9 mg/kg ΣPCBs mg/kg lipid (see [35]). The threshold was derived by Kannan et al. [34] and is based on experimental studies of both immunological and reproductive effects in seals, otters, and mink. Using thresholds in this way warrants caution owing to possible differences in species sensitivities, though it should provide a benchmark for interpreting whether associations between reproductive activity and PCB exposure are biologically significant [18]. It should be noted though that threshold levels from which effects are to be expected would be lower in embryos and calves than in adults [36].

### Multinomial logistic regression model

To further investigate the effects of ΣPCBs on reproduction, we used a multinomial logistic regression model with mature female reproductive status (1 = resting mature, 2 = pregnant, 3 = lactating) as the dependent variable and ΣPCBs (mg/kg lipid) as a covariate predictor. In addition, various other predictors or confounding factors, including both categorical and continuous, were included in the model. These comprised of ΣDDT (mg/kg lipid), region (1 = England, 2 = Wales, 3 = Scotland), year, season (season 1 = April-September, season 2 = October-March), age (years), corpora scar number (index of reproductive activity), cause of death (0 = infectious disease, 1 = trauma; proxy for health status), and three proxies of nutritional status. These included percentage of hexane extractable lipid (%lipid), ventral blubber thickness (mm), and body length-to-girth ratio—the latter was reported to be a significant predictor of survival in post-released (live-stranded) common dolphins [37]. All data were tested to establish if they violated assumptions of the multinomial logistic regression; though this type of regression does not assume normality, linearity, or homoscedasticity. A backward stepwise regression method was employed with interactive effects as oppose to a forward stepwise, as the latter is more likely to exclude predictors involved in suppressor effects [38]. The likelihood ratio test statistic was used to compare the fit of two models, one of which was the null model. Statistical analyses were performed using SPSS version 22.0 and SigmaPlot version 10.0 (SPSS Inc., Chicago, IL, USA).

## Results

Female harbour porpoises were collected between 1990 and 2012 by the UK Cetacean Strandings Investigation Programme and the UK Bycatch Observer Scheme in England (n = 123), Scotland (n = 117) and Wales (n = 89). The dataset comprised of 329 individuals for which blubber tissue was assessed for quantification of hexane extractable lipid and 25 individual lipid normalised polychlorinated biphenyls. A sub-set, 175 females, was assessed for three lipid normalised organochlorine pesticides. Within the whole dataset, females died from a range of infectious diseases (n = 134) including generalised bacterial infection, (meningo)encephalitis, pneumonia (parasitic, mycotic and bacterial) and gastritis and/or enteritis. In addition to this there were 146 cases of death due to physical trauma and 49 “other” COD cases, mostly being starvation (n = 22) and live stranding (n = 10).

### Estimation of reproductive parameters

**Fig 1:**
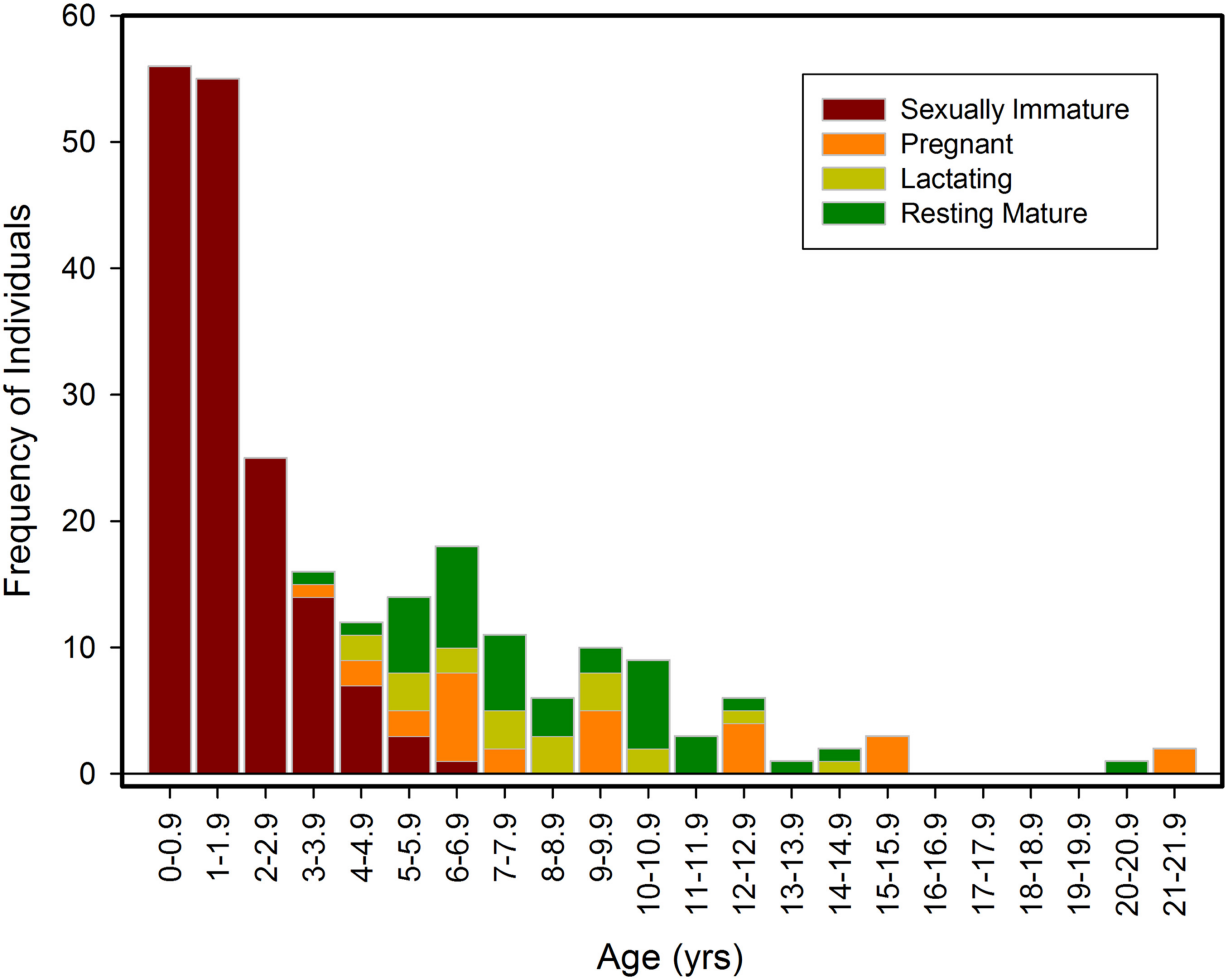
Frequency distribution of age profiles for female reproductive status categories in UK stranded harbour porpoises (n = 250).

The sample was composed of 190 sexually immature and 130 sexually mature females, and for nine individuals maturity status was not determined. Sexually immature females ranged in length and age from 70 to 157 cm (n = 187) and 0 to 6 years (n = 161), respectively, and sexually mature females from 138 to 191 cm (n = 130) and 3 to 21 years (n = 90), respectively (see Fig 1). Mean ages of sexually immature and mature females were 1.34 and 8.4 years, respectively (Table 1). Using all the dataset, the average age of females at attainment of sexual maturity was 4.73 years (standard error (SE) = 0.03, n = 250), compared to 4.63 years (SE = 0.08, n = 99) within the infectious disease group and 4.92 years (SE = 0.02, n = 114) within the trauma group.

**Table 1:** Mean age (yrs) and mean ΣPCBs (mg/kg) for the different reproductive status categories and cause of death groups.

	Sexually immature	Sexually mature	Resting	Pregnant	Lactating
**Mean age (yrs) All data**	1.34	8.40	8.09	9.18	7.66
	0–6	3–21	3–20	4–21	4–14
	(n = 160)	(n = 90^1^)	(n = 41)	(n = 28)	(n = 20)
Infectious disease	2	8.24	7.88	9.27	8.14
	0–5	3–21	3–13	4–21	4–14
	(n = 52)	(n = 47)	(n = 29)	(n = 11)	(n = 7)
Trauma	1	8	8	8.93	7.69
	0–6	3–21	4–14	3–21	5–12
	(n = 78)	(n = 36)	(n = 9)	(n = 15)	(n = 12)
Other COD	0.31	10	10.33	10.5	4
	0–3	4–20	5–20	6–15	4
	(n = 30)	(n = 7^1^)	(n = 3)	(n = 2)	(n = 1)
**Mean ΣPCBs (mg/kg) All data**	14.03	13.26	18.53	6.0	7.49
	0.48–159.68	0.4–138.83	0.95–138.83	0.74–24.76	0.4–33.13
	(n = 190)	(n = 130^2^)	(n = 60)	(n = 37)	(n = 30)
Infectious disease	17.64	15.94	20.32	6.71	9.41
	1.63–86.99	0.95–138.83	0.95–138.83	1.03–21.9	1.42–21.4
	(n = 62)	(n = 68^1^)	(n = 43)	(n = 15)	(n = 9)
Trauma	9.47	6.96	8.5	5.83	7.43
	0.48–44.15	0.74–33.13	1.92–15.37	2.34–24.76	1.29–33.13
	(n = 92)	(n = 50)	(n = 12)	(n = 20)	(n = 18)
Other COD	19.49	24.37^3^	28.02	2.33	2.03
	1.81–159.68	0.40–138.75	4.09–78.29	1.52–3.13	0.4–5.0
	(n = 36)	(n = 12)	(n = 5)	(n = 2)	(n = 3)

^1^Includes one female that was not assigned to a mature reproductive status category.

^2^Includes three individuals that were not assigned to a mature reproductive status category.

^3^Includes two females that were not assigned to a mature reproductive status category.

Using data obtained outside the conception period for the species in UK waters, pregnancy rates in the sample ranged from 26.5% for females that died from infectious disease to 50% for “healthy” individuals that died of trauma (e.g. bycatch, bottlenose dolphin kills or ship-strike) (see Table 2). However, there was no significant difference in the proportion of pregnant individuals between the infectious disease and trauma COD groups (χ^2^ = 3.520, df = 1, p = 0.0.061, n = 69). Cases of dystocia or stillbirth (cause of death) were not included in the trauma group within this analysis though for comparisons with other studies, females presenting with cases of “foetal death” were regarded as pregnant.

**Table 2:** Estimated pregnancy rates (PR: percentage pregnant) using all data, and separately for cause of death (COD) categories. PR was also estimated using data that was obtained outside the conception period (May to September [63]; see methods).

	Mature	Pregnant	PR
	(n)	(n)	(%)
**All data**	127	37	29
**All data minus conception period**	76	26	34
**COD infectious disease**	68	15	22
**COD infectious disease minus conception period**	49	13	26.5
**COD trauma**	42	13	31
**COD trauma minus conception period**	20	10	50

**Fig 2:**
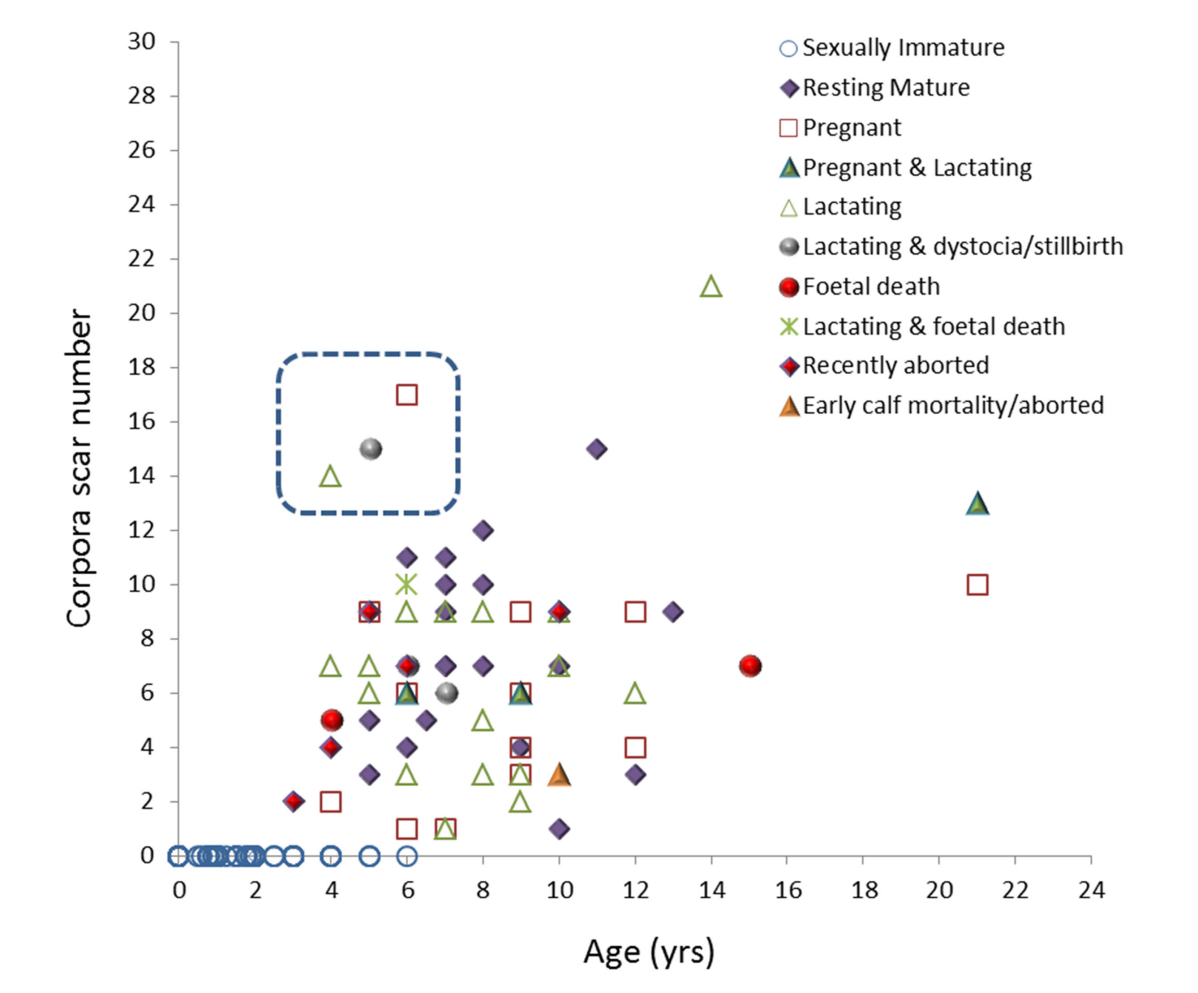
Total number of corpora scars (corpora albicantia and corpora lutea) in UK stranded harbour porpoise ovaries as a function of age (n = 225). Dashed box highlights three females that presented with higher than expected number of corpora scars.

Using number of corpora scars as an index of reproductive activity (i.e. number of ovulations), a significant positive relationship was observed between (natural logarithms transformed) age and number of corpora scars (p = 0.000, n = 225). Large-scale individual variation was observed in corpora scar number at a given age, and three females in particular presented with higher than expected corpora scar number for their age (see Fig 2). These individuals died of starvation (SW1997/178, lactating and mild multifocal eosinophilic endometritis, 4 years of age and presented with 14 corpora scars), dystocia or stillbirth (SW1997/94, lactating and pregnant, 5 years and 15 corpora scars) and infectious disease (SW2003/219a, pregnant, 6 years and 17 corpora scars).

### Evidence of reproductive failure and abnormalities

Within the whole sexually mature dataset (n = 127; for three individuals the reproductive tracts were not fully assessed), 19.7% showed evidence of reproductive failure. These include cases of dystocia and stillbirth (n = 7), foetal death (n = 4), recent abortion (n = 12), and either the female recently aborted or her calf did not survive (n = 2) (see Table 3 for an outline of definitions for these categories).

**Table 3:** Criteria for assessing categories of reproductive failure in UK harbour porpoises.

Observed categories of reproductive failure	Criteria for determining categories of reproductive failure
**Dystocia and stillborn**	Mature female died during a difficult or abnormal birth indicated by one or more of the following factors: (partially) dilated cervix; significant haemorrhage of the vagina; acute septic metritis; uterine rupture; rupturing and expulsion of the placental membranes prior to delivery of the calf; foetus was not correctly positioned; foetus was in breech presentation and it showed very marked autolytic change indicating that the calf may have died in utero and the mother had been trying to abort the body; corpus luteum showed evidence of regression on histological assessment.
**Foetal death**	Foetus was mummified or showing very marked autolytic change compared to its mother, both on gross and histological examination; and corpus luteum showed evidence of regression on histological assessment.
**Recently aborted**	Mature female died outside the conception/calving period for the population and was recently gravid based on the state of the reproductive tract such as: cervix was (slightly) dilated; uteri not fully involuted/asymmetrical uterine horns; endometrium was hyperplastic/gross or histological evidence of lesions or remodeling in the uterine body; and the presence of a regressing corpus luteum. In some cases mammary glands showed only a minimum degree of lactation, or a complete lack of activity.
**Aborted or her calf did not survive**	Mature female was recently gravid based on the state of the reproductive tract tissues and a regressing corpus luteum (as above). Mammary glands only showed a minimum degree of lactation, a lack of activity or showed evidence of “drying up” based on histological assessment. The mature female had a high PCB burden, and thus did not successfully offload her pollutant burden suggesting either abortion or early calf mortality.

Cases of infectious disease of the reproductive tract and mammary gland tissue (12 of 127, 9.5% of the total mature group) included acute suppurative endometritis in a recently aborted animal and a *Brucella ceti* infection of the mammary glands (mastitis) in another female that either aborted or whose calf died. Additionally, cases of reproductive tract and mammary gland infections included endometriosis (bacterial, mild multifocal eosinophilic; n = 2), vaginitis (n = 1), lesions of the vagina, uterus and clitoris (n = 3), a uterine tear secondary to peritonitis (n = 1), pyometra (n = 1), mastitis (n = 1), and finally one 11-year old female porpoise presented with vaginal (possibly leiomyoma) tumours, purulent metritis and reduced uterine lumen.

Tumours of the reproductive tract constituted 10.2% (13 of 127) of the total sample. Three malignant tumours (2.4% of the total mature sample) confirmed by histopathology included a squamous cell carcinoma of the cervix in a recently aborted animal (n = 1), a uterine adenocarcinoma (n = 1) and a metastasising adenocarcinoma (n = 1). Benign tumours (n = 10) included clitoral or vaginal papilloma-like lesions of suspected but unconfirmed viral cause (n = 6), vaginal epithelial plaques (n = 2), vaginal wall leiomyoma (n = 1), and, as noted above, an 11-year old female presented with vaginal (possibly leiomyoma) tumours (n = 1).

Reproductive dysfunction was also observed in 25% of individuals that should be more representative of the extant population. In the “healthy” mature female group that died as a result of trauma (n = 20) and exhibited a pregnancy rate of 50%, one female had recently aborted while foetal death was observed in another individual; and cases of uterine adenocarcinoma, papillomatous lesion of the clitoral epithelium, and vaginal epithelial plaques possibly of a viral aetiology were reported in three other individuals.

### Effects on reproduction from exposure to organochlorines

#### Offloading and accumulation of PCBs

**Fig 3:**
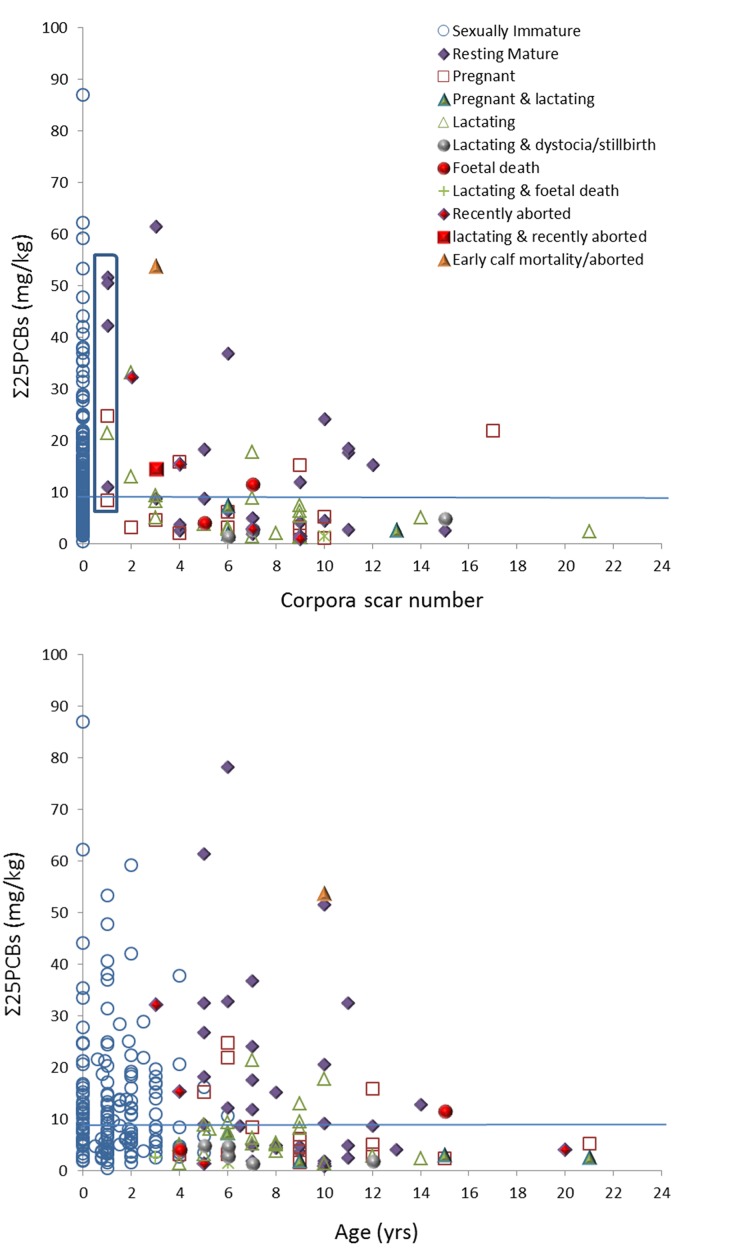
ΣPCBs as a function of (a) corpora scar number (n = 266) and (b) age (n = 254). Graphs exclude two neonates ranging between 86.9–159.7 mg/kg in ΣPCBs. Blue line represents the threshold level (9 mg/kg ΣPCBs mg/kg lipid) for adverse health effects.

Levels of ΣPCBs in sexually immature and mature females ranged from 0.48 to 159.68 mg/kg (n = 190) and 0.40 to 138.83 mg/kg (n = 130), respectively. Mean levels in sexually immature (nulliparous) and mature females were 14.03 and 13.26 mg/kg, respectively (Table 1). Females presenting with one corpora scar (corpus albicans or lutem) ranged from 6 to 10 years (n = 4) in age (Fig 2) and 144–161 cm (n = 7) in length. Mean ΣPCBs in primiparous females, including one recently pregnant and lactating female and two pregnant females, was 18.18 mg/kg (range: 8.4–24.76 mg/kg) (Fig 3a). Four resting mature females presenting with one corpus albicans scar ranged from 10.98 to 51.59 mg/kg in ΣPCBs, and where the uteri were assessed for evidence of previous gravidity (n = 3), all were previously gravid. A significant negative relationship was observed between ΣPCBs and corpora scar number in sexually mature females (p = 0.000, n = 77) (Fig 3a).

Of the aged sample, 98% were females ≤15 years and a significant negative relationship was observed between ΣPCBs and age (p = 0.003, n = 254; Fig 3b). This suggests that after offloading their pollutant burdens, female harbour porpoises do not re-accumulate PCB burdens similar to that of their pre-parturient levels in later years. All three individuals aged between 20 and 21 years were either currently or recently reproductively active, and ΣPCBs ranged from 2.65–5.15 mg/kg.

**Fig 4:**
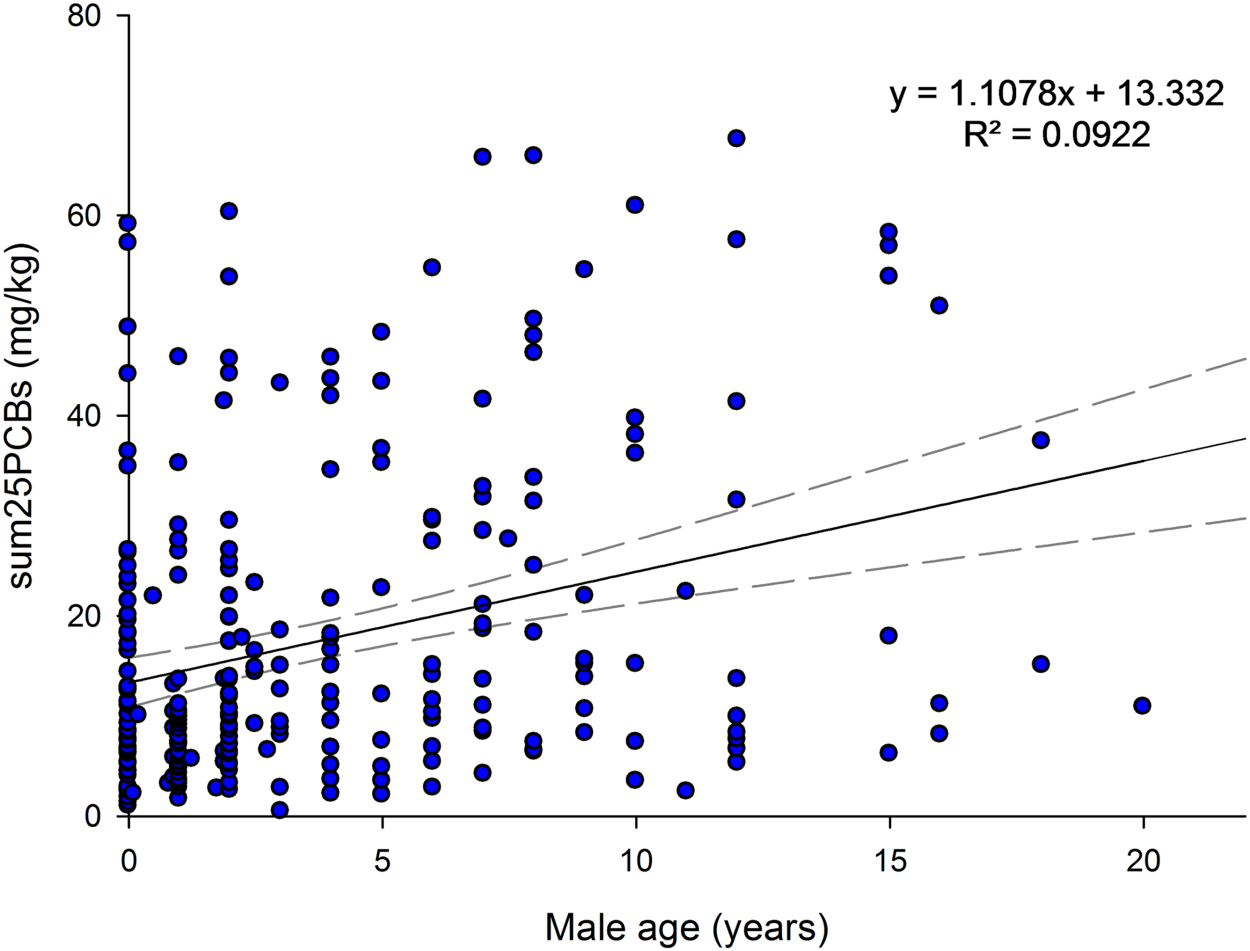
Accumulation of PCBs with age in male stranded UK harbour porpoises. Fitted regression line and 95% CI values are shown, n = 257. Annual ΣPCB accumulation rate is 1.11 mg/kg.

The relationship between male blubber PCB concentrations and age is shown in Fig 4. The least-squares linear regression equation suggests an annual accumulation rate of 1.11 mg/kg: ΣPCBs (mg/kg) = 1.11 × age (years) + 13.3, p<0.0001, R^2^ = 0.09, n = 257. The SE of the slope was 0.22.

#### Association between PCBs and female reproductive status

**Fig 5:**
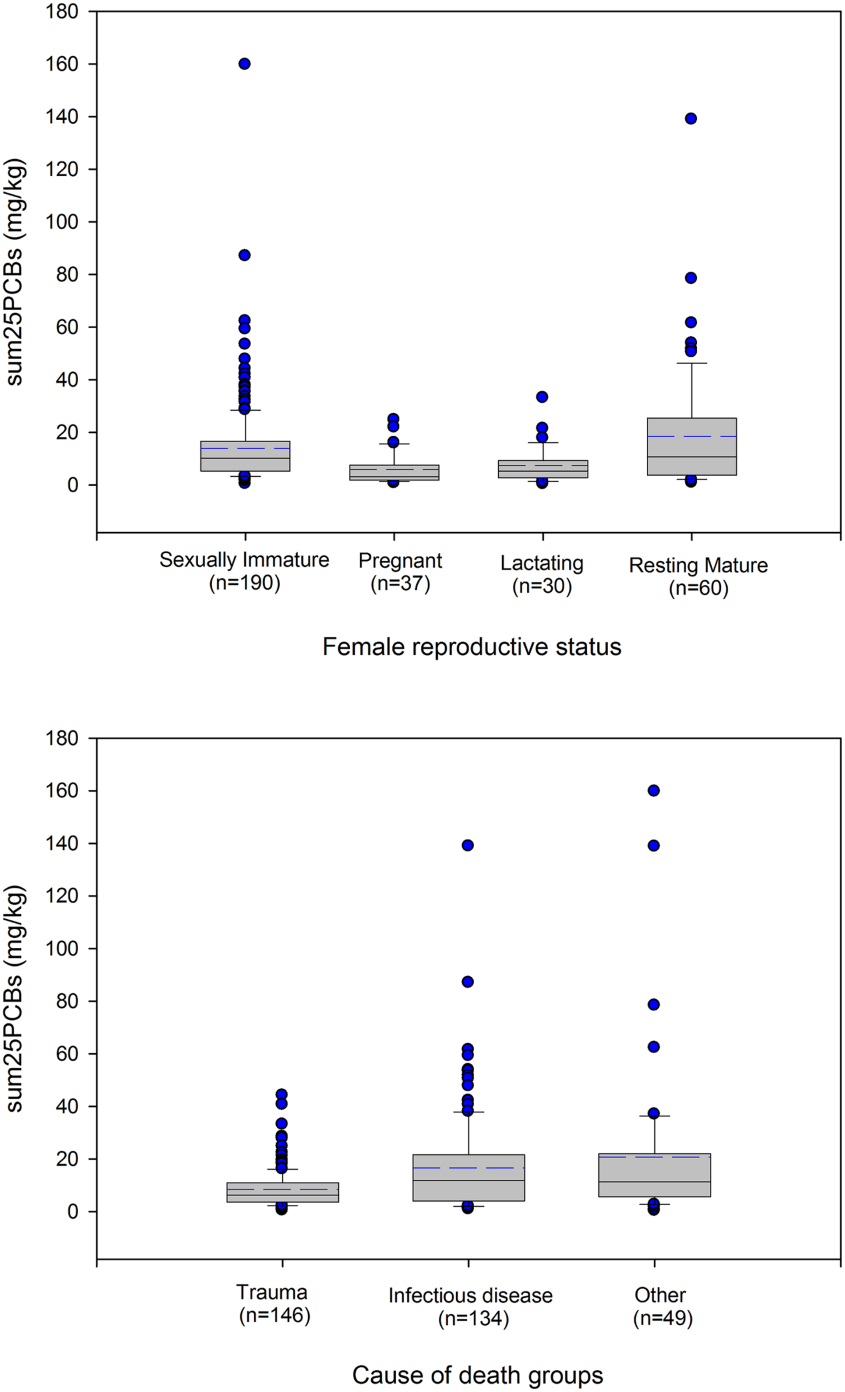
Box plots of (a) female reproductive status and ΣPCBs, and (b) cause of death groups and ΣPCBs. The dark horizontal line indicates the median, and the dashed blue line the mean. Outliers are highlighted by blue circles.

Within the whole female porpoise sample, highest mean values for ΣPCBs were reported in resting mature (18.5 mg/kg) females followed by sexually immature (14.03 mg/kg), lactating (7.49 mg/kg) and pregnant (6 mg/kg) individuals (see Fig 5a; Table 1). Using a general linear model with Tukey’s post-hoc test, a significant difference in mean ΣPCBs was observed among reproductive status categories (p = 0.000, n = 317), with the resting mature and sexually immature females having significantly higher concentrations compared to the other two groups (p≤0.009). There was no significant difference in mean ΣPCBs between sexually immature and resting females (p = 0.977). This suggests that some resting mature females had not previously offloaded as females were either nulliparous, infertile or suffered from reproductive failure. Where corpora scar data were available and excluding individuals that presented with only one corpora scar, i.e. individuals that may suffer from first-time reproductive failure, the mean values for ΣPCBs in resting mature females was only reduced to 17.1 mg/kg. Of the whole female contaminant sample 47% had ΣPCBs burdens above the threshold for adverse health effects in marine mammals (see Fig 3), and the majority of these were sexually immature (68%) and resting mature females (21%). Within the different female reproductive status categories, 52% and 53% of sexually immature and resting mature females had ΣPCBs burdens above this threshold, respectively.

The average ΣPCBs concentration in sexually immature three and four year olds (i.e. females approaching sexual maturity) was calculated. If sexually mature female harbour porpoises successfully offloaded at least 70% of their PCB pollutant burden to their first born offspring via gestation and primarily lactation [29, 30], ΣPCBs concentrations in these reproductively successful females could be as low as 3.3 mg/kg based on an average burden of 11 mg/kg (range 2.53–37.70 mg/kg, n = 22) in females approaching sexual maturity. Using an annual ΣPCBs accumulation rate of 1.1 mg/kg (Fig 4), it would take approximately 7 years before a female who successfully offloaded (approx. 70%) would re-accumulate levels above the average burden in females approaching sexual maturity. As such a large variation was observed in ΣPCBs in sexually immature females (see Figs 3 and 5), it is proposed that if sexually mature females had ΣPCBs levels above the 11 mg/kg average concentration in females approaching sexual maturity, then these mature individuals should be regarded as nulliparous, or suspected of infertility or reproductive failure.

Within the whole mature sample, 42 of 127 (33%) presented with ΣPCBs levels above 11 mg/kg. These consisted of 6 lactating, 7 pregnant and 29 resting mature female harbour porpoises. All lactating female porpoises died between March and August, i.e. the calving period, and thus did not have sufficient time to offload their ΣPCBs pollutant burden to their nursing calves. Ovarian corpora scar number in lactating females ranged from 1–7 (n = 5), and where data were available animals were 7 to 10 years (n = 3) in age. One lactating female had recently aborted and another presented with a vaginal epithelial plaque of possibly viral aetiology.

Of the seven pregnant females with ΣPCBs concentrations above 11 mg/kg, three were single cases of foetal death, dystocia or stillbirth and possible clitoral papilloma-like lesions of suspected but unconfirmed viral cause. All seven pregnant females ranged from 1–17 in ovarian corpora scar number and were 6–15 years old. The oldest pregnant female was a foetal death case with a ΣPCBs concentration of 11.6 mg/kg (Fig 3b). The individual with the highest ovarian corpora scar number was only six years in age, had a ΣPCBs concentration of 20.9 mg/kg and died of pneumonia. Although all these seven females were pregnant at the time of death, ΣPCBs concentrations suggest that they were never reproductively successful in the past.

Within the resting group 29 of 60 (48%) had ΣPCBs levels above 11 mg/kg. Of these, three recently aborted. A fourth either recently aborted or its newborn calf did not survive and also had a *Brucella ceti* infection of the mammary gland. Six other resting females (above the 11 mg/kg level) had a range of lesions including a benign leiomyoma in the vaginal wall, bacterial endometritis, vaginal papilloma-like lesions, a vaginal epithelial plaque of possibly viral aetiology, a vaginal wall lesion, and vaginitis. Finally one female (SW1990/94), who died from pneumonia, had vaginal tumours (suspected leiomyomas) in addition to purulent metritis and reduced uterine lumen. Although corpora scar number and age ranged from 1–12 and 3–14 years, respectively, the high PCB burdens (>11 mg/kg) suggest that these 29 resting females never successfully reproduced.

#### PCB levels in previously gravid females

Within the whole resting mature sample, 36 of 60 (60%) were assessed for evidence of previous gravidity on necropsy. All showed evidence of previous gravidity (100%). Ovarian corpora scar number ranged from 1–11 (n = 24). These 36 females ranged in ΣPCBs concentrations from 0.95–138.83 mg/kg and 55% of the sample was above the 9 mg/kg threshold—for these individuals corpora scar number ranged from 1–11. This suggests that high contaminant burdens, above the threshold for adverse health effects in marine mammals, did not inhibit ovulation, conception or implantation in female harbour porpoises. Additionally, as 50% of the previously gravid sample had ΣPCBs concentrations above 11 mg/kg level (mean level of sample = 39.29 mg/kg), it can be concluded that these females probably did not offload their contaminant burdens due to either foetal or newborn mortality. Females with ΣPCBs concentrations above 11 mg/kg (n = 18) ranged in ovarian corpora scar number and age from 1–11 (n = 12) and 1–10 years (n = 10), respectively. Within the whole previously gravid sample, 4 of 36 (11.1%) cases were either recent abortion (n = 3), or early calf mortality or recent abortion (n = 1).

#### Association between PCBs and causes of death in females

The highest ΣPCBs concentrations in the whole female sample (159.68 mg/kg) was measured in a neonate (SW1995/102) that died of starvation/hypothermia and stranded on the east coast of England in 1995 (Table 1). All female porpoises with pollutant burdens above 30 mg/kg (n = 33) died from either infectious disease or other non-trauma causes of death. These females ranged from 0–14 years in age (n = 26), though 73% of individuals were ≤ 5 years; of which 83% were sexually immature. The animal (SW1991/14) with the highest ΣPCBs concentration (138.83 mg/kg) within the sexually mature group was 14 years old and died from starvation.

Analysis of the data for the three COD groups showed higher mean ΣPCBs concentrations were obtained for females that died as a result of “other” causes (20.7 mg/kg, range = 0.4–159.7 mg/kg), compared to infectious disease (16.62 mg/kg, range = 0.95–138.8 mg/kg) and trauma (8.45 mg/kg, range = 0.48–44.15 mg/kg), though median values were similar between the infectious disease (11.88 mg/kg) and the “other” (11.41 mg/kg) groups (see Fig 5b). Using a general linear model with Tukey’s post-hoc test, a significant difference in mean ΣPCBs was observed among COD groups (p = 0.000, n = 329), with the trauma group having significantly lower concentrations compared to the infectious disease and the “other” groups (p≤0.001). Individuals that died from starvation constituted 22 of 49 (45%) of the “other” group. Since these females had mobilised their blubber fat reserves prior to death, this probably concentrated some of the less easily mobilised fat soluble PCB congeners [39].

#### Multinomial logistic regression

Only females that died of either trauma or infectious disease were retained within the subsequent analysis, due to the small sample size of the “other” group. Prior to undertaking regression analysis, data were checked to see if they violated assumptions of multinomial logistic regressions. Both ΣPCBs and ΣDDTs showed strong multicollinearity (VIF >10), and thus the variable ΣDDTs was removed from the analysis. The remaining variables did not violate assumptions of sample size, multicollinearity or outliers. All collinearity tolerances were low >0.55 and correlation coefficients were <0.70. The standard deviation of residuals ranged between -2 to 2, and maximum Mahalanobis distances were not greater than the critical value of χ2 for P < 0.001.

ΣPCBs was a significant factor in predicting female reproductive status (resting mature, pregnant and lactating). A multinomial logistic regression was statistically significant against the null model (χ2 = 17.558, df = 2, p = 0.000, n = 117), with 15.9% of the variance in the dependent variable explained. Analysis of predicted accuracies misclassified all lactating females as either resting or pregnant. Resting females were more prone to have higher levels of ΣPCBs than pregnant (Odds Ratio (OR) = 0.914, 95% CI = 0.859–0.971, p = 0.004) or lactating females (OR = 0.947, 95% CI = 0.901–0.955, p = 0.033). Results suggested that if the ΣPCBs were to increase by 1 mg/kg then the multinomial log-odds of a female being in a pregnant or lactating state as opposed to resting would be expected to decrease by 8.6% and 5.3%, respectively.

A backward stepwise multinomial logistic regression was employed to assess the effects of all other factors, in addition to ΣPCBs, on female reproductive status. There was evidence of effects from nutritional status (length-to-girth ratio), health status and season. Parameters such as region (i.e. country), year, reproductive activity (proxied by number of corpora scars) and other proxies for nutritional status (%lipid and ventral blubber thickness) were not found to be significant. Overall, nutritional status (length-to-girth ratio) was the most important factor in predicting female reproductive status, followed by health status (proxied by cause of death), ΣPCBs and season (Table 4). The model only including these four variables was statistically significant against the null model (χ^2^ = 63.53, p = 0.000, df = 8, n = 113), and proved a better fit with 50% of the variance in the dependent variable explained. There was no significant interaction among variables. After adjusting for the effects of confounding variables, ΣPCBs remained a significant predictor of female reproductive status (p = 0.019). Analysis of predicted accuracies suggested that the model correctly classified 66% of the sample, with the “pregnant” group being amongst the highest percentage correctly classification at 74%, though lactating females were still misclassified by 57.7%.

**Table 4:** Results from the multinomial logistic regression likelihood ratio tests including significant predictors.

Predictors		Likelihood Ratio Tests		
	Model Fitting Criteria	Chi-Square	df	Sig.
**LN:G ratio**	208.484	32.848	2	0.000
**COD class**	184.569	8.933	2	0.011
**Σ25PCBs**	183.572	7.936	2	0.019
**Season**	183.581	7.945	2	0.019

Length-to-girth (LN:G) ratio (proxy for nutritional stress), cause of death (COD) class (infectious disease and trauma; proxy for health status), ΣPCBs (mg/kg), and season (season 1 = April to September, and season 2 = October to March).

Main effects were observed for nutritional status, with pregnant females more likely to have a lower length-to-girth ratio, i.e. “fatter”, relative to both lactating (regression coefficient B = -13.232, SE = 3.02, p = 0.000) and resting females (regression coefficient B = -9.768, SE = 2.60, p = 0.000). No significant difference was observed among lactating or resting females in nutritional status. Results further suggested that females that died of infectious disease were less likely than trauma cases to be in a lactating state as opposed to resting (regression coefficient B = -1.837, SE = 0.667, p = 0.006) or pregnant (regression coefficient B = -1.685, SE = 0.774, p = 0.03). There was no significant difference among resting or pregnant females in this parameter. Season was significant as lactating females were more likely to be sampled during season 1 (April to September), i.e. during the breeding period, than Season 2. The relationship between ΣPCBs in resting females compared to pregnant (p = 0.065) and lactating (p = 0.066) females was almost significant, having controlled for other factors. With every 1 mg/kg increase in ΣPCBs the multinomial log-odds of a female being in a resting state as opposed to pregnant or lactating increased by 6.8% and 5.7%, respectively—though in both cases the 95% CI for the odds ratio included the null (1.00).

Including age in the model reduced the available sample to 79. Age was not found to be a significant predictor of female reproductive status (p = 0.252) and also, the final model did not include cause of death (proxy for health status). However, this may be a reflection of a reduction in sample size. Nutritional status remained a highly significant predictor (p = 0.000), followed by ΣPCBs (p = 0.013) and season (p = 0.022). The backward stepwise multinomial logistic regression model was run again, substituting ΣDDTs for ΣPCBs, and including variables nutritional status (length-to-girth ratio), health status and season. The model was significant (p = 0.000), though as the sample size was reduced to 64 females this violated the assumptions of logistic regression. Three variables were retained in the model, including nutritional status (p = 0.001), health status (p = 0.009) and ΣDDTs (p = 0.042).

## Discussion

This study encompasses the largest dataset produced to date on PCB burdens in female harbour porpoises within a specific population. The study analysed data and samples collected over a 22-year period from one of the longest running international stranding programmes, thus providing the best opportunity to assess evidence of reproductive failure in this elusive marine species.

### Estimation of reproductive parameters

A pregnancy rate of 34% and an average age at attainment of sexual maturity of 4.73 years were estimated using all available data within the current study. Data arose from samples obtained by the UK Cetacean Strandings Investigation Programme and the UK Bycatch Observer Scheme. Using these types of data can cause biases, which may subsequently lead to erroneousness estimation of population reproductive parameters. Primarily, as the stranded sample may be composed of ill health and/or poor condition individuals, and also older individuals with reduced reproductive capacity/fertility. Whereas porpoises in this region exhibiting a strong susceptibility to incidental capture in fishing gear are predominately weaned juveniles and sub-adults [40]. For some species, such as the common dolphin in the NE Atlantic, stranded animals have been found to be representative of the population and used to estimate reproductive parameters [41]. Primarily because the mature stranded sample was heavily composed of bycaught “healthy” dolphins (i.e. did not die of infectious disease) that washed up on European shores and also females that died during mass live stranding events. However, given the high number of diseased porpoises within the current study, it is not known if the stranded harbour porpoise sample is really representative of the extant population.

The large numbers of diseased porpoises that strand on a yearly basis through-out the North-east Atlantic, North and Baltic Seas [18, 23, 42, 43] would suggest a higher prevalence of diseased porpoises in this region. This has been substantiated by studies revealing a poor general health status of porpoises inhabiting the North and southern Baltic Seas compared to more northern regions. Siebert et al. [44] reported that bycaught and hunted harbour porpoises from Greenlandic, Icelandic and Norwegian waters were healthier than bycaught porpoises from the North and Baltic Seas. Overall lesions associated with parasitic infections were milder and severe secondary bacterial infections were infrequent. A follow up study assessed the regional differences in bacterial flora in harbour porpoises within the North-east Atlantic by sampling bycaught, hunted and stranded animals over an 18-year period. Significantly less bacterial growth and fewer associated pathological lesions were observed in porpoises from Icelandic and Greenlandic waters, compared to animals inhabiting the North Sea, Baltic Sea and Norwegian waters [45]. These observed differences were attributed to possibly impacts from stressors resulting from anthropogenic activities, such as exposure to chemical pollutants, in North and Baltic Sea porpoises.

Nevertheless, reproductive parameters were also estimated using a sample of “healthy” porpoises that died as a result of a major trauma (incidental capture, bottlenose dolphin kill and ship strike). What is evident from Table 5 is that harbour porpoises in UK waters have a substantially lower pregnancy rate (50%) and a higher average age attained at sexual maturity (4.9 years) compared to harbour porpoises in other geographic areas, such as the Gulf of Maine and Bay of Fundy in the North-west Atlantic (93%, 3.27 years) [46], and waters off Iceland (98%, 3.2 years) [47]. Previously, Read and Gaskin [48] proposed that the North-west Atlantic population was exhibiting density dependent compensatory responses due to high bycatch rates in sink gillnet fisheries. Female harbour porpoises sampled between 1985 and 1988 attained sexual maturity at a significantly younger age and shorter length than females sampled between 1969 and 1973. Mean calf length also significantly increased in size between these two periods. However, it has also been suggested that in addition to bycatch, changes in the growth responses of porpoises in the Bay of Fundy/Gulf of Maine were strongly coupled with changes in the availability of herring and their energy content [49]. Either way, what was apparent from the North-west Atlantic reproductive studies were that most females were on an annual reproductive cycle and as a high proportion of lactating females were sampled, especially in the early summer, this indicated that most pregnancies resulted in the birth of a viable calf [46]. Due to a lack of historical biological data for porpoises off Iceland¸ it is not known if the porpoises sampled during the 1990s, with a high reproductive output, were exhibiting density dependent and compensatory effects [47].

**Table 5:** Estimated pregnancy rates, based on the presence of a foetus and excluding the conception period, of mature female harbour porpoises.

Country/Region	Sampling period	Pregnancy rate	n	ASM (yrs)	n	Reference
**UK (All stranded and bycaught porpoises)**	1990–2012	0.34	76	4.73	250	Current study
**UK (Trauma sample)**	1990–2012	0.50	20	4.92	112	Current study
**Scotland**	1992–2005	0.34	29	4.35	144	[63]
**North-east Atlantic** ^1^	2001–2003	0.42	26			[20]
**Denmark** ^2^	1985–1991	0.73	33			[50]
**Sweden(mainly Kattegat Sea)**	1988–1990	0.67	18			[118]
**Iceland**	1991–1997	0.98	74	3.2^3^	269	[47]
**Newfoundland and Labrador, North-west Atlantic**		0.76				[119] reported in [120]
**Bay of Fundy, North-west Atlantic**		0.90	10			[121] re-analysed data from [122]
**Bay of Fundy, North-west Atlantic**	1985–1988	0.74	35			[121]
**Gulf of Maine, Bay of Fundy, North-west Atlantic**	1989–1993	0.93	14	3.27^4^	11	[46]
**Massachusetts, North-west Atlantic** ^5^	1975–1989	0.72	18			[123]

^1^Included porpoises from the Iberian Peninsula population, and all causes of death.

^2^Included porpoises from the Danish North Sea and Inner Danish waters.

^3^Average age of 50% mature.

^4^Mean age of first-time ovulators.

^5^Study did not exclude the mating period, and included two recently pregnant lactating females.

Within the NE Atlantic and contiguous seas, Sorensen and Kinze [50] estimated a pregnancy rate of 73% for Danish waters. This pregnancy rate was estimated using samples obtained from both the Danish North Sea and inner Danish waters. The latter region is encompassed within the geographic distribution of the recently defined western Baltic Sea (Kattegat and the Belt Seas) subpopulation that has an abundance of 40,475 (CV = 0.235) porpoises and is classified as “Vulnerable” on the HELCOM Red List [51, 52]. An abundance of 375,358 (CV = 0.197) porpoises was estimated for the EU Atlantic continental shelf, North Sea and western Baltic Sea waters in July 2005, and there was no evidence that total harbour porpoise abundance varied between then and (areas surveyed during) an earlier survey undertaken in July 1994 [53]. However, the status of harbour porpoises in the NE Atlantic and North Sea in 1994 is unknown, including whether the abundance estimate was reflective of a depleted population or a population close to carrying capacity. Based on evidence of large-scale incidental captures of porpoises in fishing gear prior to July 1994 (e.g. [54, 55]) it would not be expected that the harbour porpoise population in this region was close carrying capacity at that time, notwithstanding a declining suitable prey base. If the NE Atlantic population was recovering from over exploitation in the past, we would not expect the results observed in the current study, i.e. a longer calving interval, lower pregnancy rate and later maturation than other populations. Whereas, the existence of a “younger” porpoise NE Atlantic population, where most individuals do not live beyond 12-years of age ([19, 40], and the current study), is not reflective of a population at carrying capacity and is suggestive of other factors being at play.

Pregnancy rates were estimated in the current study by excluding individuals that died during the mating period, so as to avoid missing early embryos. Undertaking this approach also excludes cases of abortion that may occur during very early gestation, though not those that occur later. Thus, the birth and fecundity (incorporating newborn survival to a certain date) rates will ostensibly be lower.

### Evidence of reproductive failure and abnormalities

19.7% of mature females showed direct evidence of reproductive failure (foetal death, aborting and dystocia or stillbirth) in the current study. In addition, a further 16.5% had infections of the reproductive tract and/or tumours of reproductive tract tissues that could contribute to reproductive failure. Ovarian abnormalities have not been discussed in the current paper, and are currently being investigated including cases of both ovarian hyer- and hypo-plasia and possible tumours (Murphy unpublished data), and thus estimates of reproductive abnormalities should be regarded as a minimum. ΣPCB profiles provided further evidence of reproductive failure within UK porpoises, with at least 48% of resting females not offloading their high pollutant burden via gestation and primarily lactation. Where data were available, these non-offloading females were previously gravid, which suggests foetal or newborn mortality. This resting sample was comprised of 25 females that did not show direct evidence of reproductive failure (as above). Thus, reproductive failure could have occurred in up to 39% or more of the mature females sampled.

Female mammals can experience high rates of reproductive failure [56, 57]. Disturbance at any point of reproduction can lead to failure, manifested in an inability to conceive or implant, abortion, premature birth, stillbirth, or illness or death of the newborn [58]. In marine mammals, reproductive failure can be caused by genetic defects, congenital disorders, systemic infection, central nervous system disease, neoplasia, ageing (senescence), nutritional or environmental stress, anthropogenic contaminants/endocrine disruptors and marine biotoxins [58–61]. Promiscuous mammal species have been found to have significantly lower rates of early reproductive failure compared to mammal species adopting other reproductive strategies and, moreover, parentage genetic incompatibility, a major potential cause of early reproductive failure has been proposed as one of the main drivers behind polyandry [56]. Thus, the deleterious fitness consequences of inbreeding can be overcome in small promiscuous populations by postcopulatory selection for dissimilar gametes, which has been proposed for the North Atlantic right whale (*Eubalaena glacialis*) [62]. Harbour porpoises, like right whales, are a promiscuous species with possibly high levels of sperm competition [63–65]. Although the exact mechanisms behind pre- and postcopulatory selection in harbour porpoises are unknown, sperm selection is likely to maintain heterozygosity and reduce instances of parentage genetic incompatibility and thus, occurrences of early reproductive failure.

Within marine mammals, reproductive failure has been reported in numerous species, most notably in pinnipeds and otariids, and predominately related to nutritional stress. Nutritionally stressed seals can either avoid reproductive costs altogether by failing to implant after embryonic diapause [66] or abort during late-gestation, a period of high energetic demands for pregnant females [67, 68]. The prevailing strategy may depend on age, as found in Australian fur seals (*Arctocephalus pusillus doriferus*). Younger fur seals showed a tendency to abort during late gestation, whereas older seals were possibly more prone to reproductive failure at the time of fertilization, implantation, or early gestation [67]. Even if seals give birth, when food resources are scarce newborn seal pups can exhibit reduced birth weights and growth rates, and both pup and juvenile survival can be low [69]. Nutritional/body condition has also influenced pregnancy rates in large cetaceans. For example, female North Atlantic fin whales (*Balaenoptera physalus*) with thin blubber layers, which occurred during periods of low *per capita* prey abundance, had significantly lower probability of being pregnant than those with average blubber thickness [70]. Reduced prey abundance has similarly been linked to lower fecundity in killer whales (*Orcinus orca*) in the North-east Pacific, with the probability of a female calving differing by 50% between years of low and high salmon abundance [71].

In the current study, although nutritional status (length-to-girth ratio) was found to be the primary predicator of reproductive status (pregnant, lactating, resting) in mature females using the multinomial logistic regression model, results were not of significance. Pregnant females were more likely to be “fatter”, compared to resting and lactating females, the same situation as in all mammals as females need to store energy to cover the additional energy requirements of lactation. Additionally, due to the location of the dorsal fin in porpoises situated further back along the dorsal aspect, the occurrence of pregnancy will more than likely have an impact on the girth of the animal as it was measured in front of the dorsal fin. No significant difference was observed among lactating or resting females in this parameter within the multinomial logistic regression model. Variations in foetal and newborn birth weights were not assessed within the current paper, which may be an area of further study. Particularly in the case of pollutant exposure, as prenatal exposure to PCBs and hexachlorobenzene were found to impair foetal growth in humans [72]. Additionally, malnutrition can delay attainment of sexual maturity, and should be considered further.

Amongst harbour porpoises, very few cases of reproductive failure have previously been documented and those that have were reported as cases of dystocia or stillbirth. Dystocia or stillbirth was suspected as the cause of death in 18 newborn harbour porpoises necropsied in Germany between 1991 and 1996 [42]. Dystocia, difficult and/or long labour, can be caused by abnormal foetal presentation and/or large foetuses, or foetal congenital abnormalities such as a deformed tailstock [73, 74]. Within the current study, all seven pregnant female harbour porpoises that died as a result of dystocia or stillbirth (18.9% of the pregnant sample) were found in June (86%), and April. Foetuses measured between 55 and 83 cm in length, which is mostly within the length-range for newborn porpoises in UK waters (60–84 cm) [63, 75]. In three instances, porpoise’s live-stranded *in extremis* whilst trying to abort or give birth to their calf, whilst in another case even though the foetal membranes had ruptured, the foetus was not correctly positioned for a normal delivery. Complications such as a large foetus and an acute necrotising metritis were associated with the remaining dystocia or stillbirth cases. For one live-stranded female, death was probably due to a combination of dystocia, uterine rupture and septic shock that may have developed as sequelae to foetal death and the establishment of acute septic metritis.

All reported cases of foetal death and recent abortion occurred between November and April, during the females second (56%) and third trimesters (44%) based on a mean date of conception of the 4^th^ August and a gestation period of 10.3 months for porpoises in Scottish waters [63]. In addition to these, there were two cases where the female either recently aborted or her newborn calf did not survive. These females died in June and September, i.e. during the calving/mating period. It should be noted that abortions during very early gestation (first trimester) may have been missed in other individuals that were lactating and recently calved. What these results show however is the occurrence of foetal death and abortions after the first trimester in UK female harbour porpoises—these cases comprised 12.6% of the mature female sample. This may be reflective of the health status of females, as 86% of the foetal death/aborting group died as a result of infectious disease or other causes such as starvation and neoplasia. Of the females that recently aborted, one individual presented with acute suppurative endometritis, and cervix squamous cell carcinoma with intra-abdominal metastastis was reported in another female that led to uterine perforation at the time of early parturition. *Brucella ceti* was identified in the mammary gland tissue of a female that either recently aborted or whose newborn calf did not survive. *Brucella* spp. are known to induced abortions in marine mammals [76], and a *Brucella ceti* was isolated in association with a possible abortion in a Belgium harbour porpoise [77].

Within the mature sample, 24 of 127 (18.9%) female harbour porpoises presented with malignant tumours, benign tumours such as leiomyoma, papilloma-like lesions and vaginal plaques, endometritis, and other infections and inflammations of the reproductive tract, all which could contribute to reproductive failure. These results are in contrast to an earlier study on stranded and bycaught harbour porpoises from the German North and Baltic Seas, where lesions of the female genital system were rare—authors noted one case of suppurative endometritis that may have impaired reproduction [42]. A subsequent study reported a granulosa cell tumour of the ovary in a 12-year old female harbour porpoise that stranded on the German North Sea coast [78]. Possible cases of clitoral or vaginal papilloma-like lesions of suspected but unconfirmed viral cause were the most prevalent abnormalities in the current study, observed in 4.7% of mature females. Papilloma viruses can be host- and tissue-specific causing epithelial and fibroepithelial benign tumours and although these lesions typically regress spontaneously, tumours may persist and progress to invasive carcinomas [79]. Three mature female harbour porpoises (2.4% of the mature sample) presented with cases of malignant tumours of reproductive tract tissues; including cervix squamous cell carcinoma (as above), uterine adenocarcinoma, and metastasising adenocarcinoma. Although occurrences of neoplasms are rare in marine mammals [80], a high cancer rate (primarily of the digestive tract) was observed in the threatened (COSEWIC status 2004) St Lawrence Estuary beluga whale population [8]. At 18%, it was the second leading cause of death amongst stranded animals—though tumours were observed in 27% of animals. The high frequency was attributed to environmental exposure to carcinogenic substances and/or reduced immune function due to limited genetic diversity [8, 80]. Within the study, adenocarcinomas were reported in the mammary gland tissue of three beluga whales and the uterus of another, and there were three reported cases of ovarian neoplasms. Other marine mammal species showing increased incidences of neoplasms include California sea lions, where a lethal metastatic genital carcinoma was reported in 18% of stranded sexually mature individuals sampled between 1979 and 1994 [81, 82]. Both a virus (Otarine herpesvirus-1) and carcinogenic contaminants were proposed putative causes for this high incidence of metastatic carcinomas of genital origin [83, 84]. It was suggested that OCs may have acted as immunosuppressive agents, or increased incidences of carcinoma through genotoxic mutation and tumour promotion [83].

The reproductive failure rate in harbour porpoises, excluding occurrences of effects from various stressors such as pollutants, is unknown. However, research has shown that most mature female harbour porpoises in a population can reproduce annually [46, 47], if conditions are suitable. A high foetal survival rate has previously been observed in bottlenose dolphins, where 83% (10 of 12) of detected pregnancies assessed during health assessments of the Sarasota Bay population (1998–2013) resulted in live births [85]. In the current study, reproductive failure may have occurred in 39% or more of mature females. Thus, effects of intrinsic and extrinsic factors on reproduction in these females, and possibly newborn calf survival, require investigation. In mammals, females may optimise their life time reproductive output, through an adaptive mechanism, by supressing reproduction when future conditions for survival of offspring are likely to be more favourable than present ones, as to exceed to costs of suppression itself [57]. Earlier terminations are physiologically easier to induce than later ones ([57] and references therein) and ultimately terminations during late gestation would incur severe health and reproductive costs, including impacting a female’s subsequent ability to reproduce, or precede death. Within the current study, reproductive dysfunction was observed throughout the study period (1990–2012), including occurrences of reproductive failure during the second and third trimesters. In a number of cases, reproductive failure, such as cases of dystocia or stillbirth, was the leading cause of maternal mortality. Although there was no significant evidence of effects from nutritional condition on reproductive status, apart from pregnant females being “fatter”, large-scale changes in prey quality availability during the last few decades due to overfishing and environmental (climate) change must have impacted population density/status and should be considered further. Particularly since individual health status of harbour porpoises in the North-east Atlantic often present “energetic knife-edge” conditions. What was evident in the dataset was that lactating females, i.e. females who successfully reproduced, were more likely to be in good health status (died as a result of trauma) compared to other females. Further, 86% of cases of foetal death and abortion were observed in females that died of infectious disease or other causes such as starvation and neoplasia. Thus, female’s health status plays an important factor in the occurrence of reproductive failure. We did not assess a number of other stressors known to impact reproduction in mammals, such as psychological and social stress [86, 87]. The effects of psychological stress should be considered further considering the scale of current and future marine renewable developments in European waters [88] and other large-scale anthropogenic activities in the marine environment that cause noise and disturbance. Social suppression of reproduction, which has been reported in numerous gregarious species [57, 86], was not considered as although group dynamics has not fully been investigated in this species, it is believed that individuals do not exist within a social hierarchical system [89].

### Effects on reproduction from exposure to organochlorines

OCs are endocrine disruptors, and the endocrine and reproductive effects of these chemicals are believed in part to be due to their ability to mimic, antagonise and disrupt the synthesis, metabolism and transport of endogenous hormones, and synthesis of hormone receptors [90, 91]. Some PCB congeners have a dioxin-like mechanism, while others are estrogenic and/or anti-androgenic, and interactions with other pollutants can produce additive or non-additive effects [91]. While metabolites of PCBs can have estrogenic, as well as anti-estrogenic effects [92].

In the current study, results suggest that PCB burdens did not inhibit ovulation, conception or implantation as, based on the state of their uteri, all examined resting female harbour porpoises with ΣPCB concentrations >11 mg/kg were previously gravid. Similar to what was reported in NE Atlantic common dolphins [26]. However, PCBs may have negatively impacted foetal or newborn survival as previously gravid resting female harbour porpoises had not successfully offloaded their pollutant burdens, via gestation and primarily lactation. Results from the multinomial logistic regression model showed that resting females were more prone to have higher levels of ΣPCBs than pregnant or lactating females. Further, 12.6% of mature females presented with evidence of foetal death or abortion during their second and third trimesters. In mink (*Mustela vison*), experimental studies have shown that PCBs increased rates of foetal mortality. This was mediated by affecting maternal vasculature in the placenta and producing degenerative changes in the trophoblast and foetal vessels that lead to foetal growth retardation or death [93, 94]. In humans, an association was also reported between PCB-153 and an increased risk of foetal loss (miscarriages or stillbirths) [95], though other studies did not observe these particular effects (e.g. [96]). Meeker et al. [96] reported that PCB-153 and ΣPCBs were associated in humans with significantly elevated dose-dependent odds of failed implantation, possibly through PCB impacting uterine receptivity. Somewhat similar to that reported in European harbour seals (*Phoca vitulina*), where hormone profiles indicated that PCB-mediated effects occurred at the stage of implantation, while follicular, luteal and post-implantation phases were not affected [1]. Estradiol-17β levels in seals fed with fish of a higher contaminant burden were lower than those of the control group, and it was reported that lower estradiol levels may have impaired endometrial receptivity and prevented successful implantation of the blastocyst [97]. Reijnders [97] hypothesized that enhanced breakdown of estradiol through enzyme-induced metabolism by PCBs was a possible mode of action.

PCB-mediated effects could also have negatively impacted (first) newborn survival of UK porpoises, a situation observed in bottlenose dolphins were PCB-related risk of stillbirths or neonate mortality from primiparous females ranged from 60–79% among three populations in the NW Atlantic [3, 98]. Further, the St Lawrence Estuary Beluga whale population showed reduced fecundity likely linked to high contaminant burdens, as proportions of calves and juveniles were lower than in populations occurring off Alaska [99]; indicating a decreased birth rate and/or increased juvenile mortality. Other work identified that milk production was compromised in 41% of stranded St Lawrence Estuary mature female Belugas [100], which may have impacted annual fecundity rates. Σ25CBs concentrations in newborn and neonate calves (<1 year) in the current study ranged from 1.8–159.7 mg/kg (n = 61, average = 16.26 mg/kg), indicating significant offloading to some (probably first-born) offspring during the gestation and lactation periods. Interestingly, the highest contaminant burden (Σ25CBs = 159.7 mg/kg) in the whole study was observed in a porpoise calf measuring 90 cm in length. This individual (SW1995/102) was reported to have been in a very poor nutritional condition, died of starvation/hypothermia soon after birth (teeth were unerupted and papillae were prominent on the tip of the tongue) and presented with congenital oesophageal stenosis, and oesophageal (and fundic stomach) ulceration. As a causal immunotoxic relationship has previously been reported between PCB exposure and infectious disease mortality in UK harbour porpoises [18], in addition to neurobehavioral and developmental deficits reported in juvenile humans exposed to PCBs in utero (e.g. [101], the risk of mortality in UK harbour porpoise calves from pre- and post-natal PCB exposure requires further investigation.

As known endocrine disruptors, early (pre- and post-natal) exposure to PCBs during a sensitive stage of development or differentiation may result in latent effects on reproductive function and sexual behaviour (i.e. the developmental basis of adult disease), in addition to generational epigenetic effects [102–104]. For example, there was a reported link between the occurrence of vaginal adenocarcinoma in women exposed in utero to an endocrine disrupting chemical [102, 105]. Endometriosis, uterine leiomyomas and other reproductive tract anomalies, have also been proposed as reproductive and endocrine toxicity endpoints in animals and humans [102, 106]. Female UK harbour porpoises ranged in blubber Σ25CBs concentrations from 0.4–4.86 mg/kg in individuals presenting with malignant tumours of the reproductive tract, 2.65–78.29 mg/kg in females presenting with benign tumours, 0.9–32.9 mg/kg in cases of endometriosis, and in the female presenting with *Brucella ceti* infection of the mammary gland it was 53.8 mg/kg. Further, not all females that aborted or whose foetuses or newborn calves did not survive within the current study presented with high PCB burdens (Σ25CB concentrations ranged from 0.69–53.81 mg/kg). However, relating the presence of these reproductive-related disorders to blubber ΣPCB concentrations in mature females would prove inconclusive, as lipid-rich milk is a major route of elimination of lipophilic PCBs in lactating females. Thus, although PCB-mediated effects may have occurred during pre- and post-natal developmental, correlations between current pollutant burdens and endpoints for reproductive toxicity would not be evident if a female had offloaded. Only in cases where there is profound reproductive impairment causing lifetime sterility, as in the Baltic seals (see below), can these associations be made. Notwithstanding, when all ages were considered 47% of individuals in the current study had ΣPCBs concentrations above the threshold for adverse health effects (the level when immune system and endocrine endpoints were affected) in marine mammals, which included 52% of sexually immature females and 53% of resting mature females.

Effects of OC exposure have been linked to cases of reproductive failure in other marine mammal species, with the most comprehensively documented effects reported in Baltic seals. The numbers of Baltic seals, both ringed (*Pusa hispida*) and grey seals (*Halichoerus grypus*), declined dramatically in the early 1900s due to hunting, and thereafter due to pollution ([107, 108], and references therein). A high prevalence of reproductive tract lesions were observed in seals sampled during the 1970s and early 80s, including occlusions and stenosis of the uterine horns (42% of sampled grey seals) and benign uterine leiomyomas (53% of grey seals older than four years), which were related to high levels of PCBs and DDT [4, 5, 109]. Initially, disentangling the effects of exposure to multiple pollutants on the prevalence of these disorders proved difficult, and relied on other published work that showed somewhat similar reproductive effects from exposure to PCBs [5]. Though more recently, an estimated exposure index indicated that PCBs, moreover than DDT, were associated with the increased prevalence of uterine leiomyomas in Baltic grey seals [4]. Although uterine obstructions were non-lethal, they rendered females partially or completely sterile for life [97]. Obtrusions may have developed as a result of PCB-induced abortions or foetal death, as some individuals retained foetal membranes aligned to stenosed sections of the uterine horns [2, 5, 110]. Purulent endometriosis was also observed in some seals presenting with obtrusions [2].

Compared to other mature females, mean ΣPCBs concentrations were higher in resting females that died of infectious disease and “other” causes of death (Table 1). Although there was no significant interaction among variables health status (cause of death) and ΣPCBs in the multinomial model, PCBs-mediated effects on reproduction could arise via immunosuppression and increased disease risk. Experimental studies have shown that PCBs suppress immune functions in marine mammals and thus, increase susceptibility to opportunistic infectious diseases caused by bacteria, viruses, or parasites ([111], and references therein). Increased susceptibility to infection from exposure to PCBs would not only compromise adult survival, but may also have consequences on uterine and placental health and, subsequently, foetal health and survival [26, 112]. Within the current study, female’s health status played an important factor in the occurrence of reproductive failure. Thus, PCBs-mediated effects on reproduction in harbour porpoises may occur via both direct and indirect mechanisms.

Unlike bottlenose dolphins [31], harbour porpoises in the current study showed a decrease in pollutant burden with age. This however does not suggest that all females rid themselves of their pollutant burden, and successfully reproduced. Within the sample, 93% (14 of 15) of mature females with ΣPCB burdens ≥30 mg/kg died as a result of infectious disease or “other” causes such as starvation, and these cause of death groups also comprised 92% (23 of 25) of the pollutant sample ≥20 mg/kg. Accordingly, female harbour porpoises with higher pollutant burdens were effectively removed from the population due to ill health. Whether or not the occurrence of pregnancy in mature individuals with higher ΣPCB burdens attributed to their ill health remains to be seen. Though for one pregnant female (SW1994/7a) who died of a severe bronchopneumonia associated with abscessation and parasitism, this may have been facilitated or exacerbated by the stress and/or immunosuppression that can be associated with pregnancy. Previous studies that produced PCB exposure simulation models suggested a growth dilution (decline) phase of PCB concentrations in sexually immature individuals; for example in bottlenose dolphins from the time they are weaned up to about 5 years of age [113]. This however is not apparent in sexually immature individuals in the current study (see Fig 3b), though requires further investigation.

This paper has focused on possible effects from PCBs and although DDT has been linked to cases of reproductive dysfunction in other species (reviewed in [114]) and was strongly correlated to PCB levels in the current and previous studies on UK porpoises [18, 115], DDT concentrations significantly declined in UK porpoises since the early 1990s [17]. Nevertheless, the additive effects from exposure to multiple pollutants cannot be ruled out, nor the effects of low exposure to DDT at sensitive developmental life stages. For instance, it has been reported that human disorders are more likely the result of chronic exposure to low amounts of mixtures of endocrine disrupting chemicals [102].

## Conclusions

We assessed for evidence of reproductive failure both directly, e.g. through observations of foetal death, and indirectly by using individual PCB burdens. Using these data, results suggested that reproductive failure could have occurred in up to 39% or more of mature females sampled. Above all, female’s health status played an important factor in the occurrence of reproductive failure. However, within the group of “healthy” UK harbour porpoises that died as a result of trauma we still observed a lower reproductive output compared to other (less contaminated) populations; with a lower pregnancy rate (almost half the rate) and a higher average age at attainment of sexual maturity observed. PCBs may have negatively impacted foetal or newborn survival as previously gravid resting female harbour porpoises had not successfully offloaded their pollutant burdens, via gestation and primarily lactation. Though ultimately difficulties arose in showing casual associations between cases of reproductive dysfunction and ΣPCBs concentrations due to female’s capabilities in offloading pollutant burdens. Nevertheless, when all ages were considered 47% of individuals in the current study had ΣPCBs concentrations above the threshold for adverse health effects in marine mammals, which included 52% of sexually immature females and 53% of resting mature females. Whether or not the stranded harbour porpoise sample is reflective of the extant population will continue to be debated. Even though previous research reported that bycaught harbour porpoises in the North Sea were in a poorer health status than their more northern counterparts [44], which may explain the high occurrence of diseased stranded harbour porpoises in this region. Interestingly, the higher pollutant levels in some resting females provided information on their lifetime reproductive success, and as a result samples from necropsied animals provided more information than a prevue of reproductive status at the time of death. Preliminary results suggest that reproductive dysfunction in UK porpoises may be related to PCB exposure occurring either through endocrine disrupting effects or via immunosuppression and increased disease risk. Declines of major organochlorine concentrations in biota have been slow due to global cycling and long-half lives of pollutants [116], and as of 2005 1.1 million ton of PCB containing equipment, corresponding to 350,000 ton of PCB containing liquid, still required disposal by EU Member States [117]. Taking this into consideration, as well as inherited maternal pollutant burdens in first born offspring and generational epigenetics effects, raises concerns about the current and future population-level effects of PCBs on the continuous-system North-east Atlantic harbour porpoise population.

## Supporting Information

S1 Dataset Female harbour porpoise contaminant data. (DOC)
