# Supplementary material for: Reproductive Failure in UK Harbour Porpoises Phocoena phocoena: Legacy of Pollutant Exposure?
Source: PLoS One. 2015 Jul 22;10(7):e0131085. doi: 10.1371/journal.pone.0131085 (PMC4511585; doi:10.1371/journal.pone.0131085)
Supplement: S1 Dataset — (DOC) [file pone.0131085.s001.doc]

| **National Ref** | **Species (Scientific)** | **Date found** | **Location** | **Region** | **Sex** | **Length** | **sum_ 25PCBs_LIP** | **sum_DDT_LIP** |
| --- | --- | --- | --- | --- | --- | --- | --- | --- |
| SW1990/39 | Phocoena phocoena | 08/04/1990 | South Penally beach (nr Giltar Pt), Tenby | West coast | F |  | 20.22 |  |
| SW1990/50 | Phocoena phocoena | 20/06/1990 | Carmarthen Bay | West coast | F | 138 | 16.74 |  |
| SW1990/71 | Phocoena phocoena | 03/09/1990 | Johnshaven Harbour (600 yds S.of), Angus | Grampian | F | 146 | 3.85 |  |
| SW1990/72 | Phocoena phocoena | 05/09/1990 | Abersoch | West coast | F | 106 | 40.62 |  |
| SW1990/84 | Phocoena phocoena | 09/10/1990 | South beach, Bridlington | East coast | F | 150 | 32.86 |  |
| SW1990/92 | Phocoena phocoena | 30/10/1990 | Ainsdale | West coast | F | 180 | 0.40 |  |
| SW1990/94 | Phocoena phocoena | 07/11/1990 | Sea Palling | East coast | F | 151 | 32.53 |  |
| SW1990/98 | Phocoena phocoena | 18/11/1990 | Westdale Beach (nr Dale) | West coast | F | 125 | 2.01 |  |
| SW1990/100 | Phocoena phocoena | 21/11/1990 | Shell Island | West coast | F | 125 | 1.63 |  |
| SW1990/107 | Phocoena phocoena | 05/12/1990 | Druidston Haven, St David's | West coast | F | 176 | 9.25 |  |
| SW1990/109 | Phocoena phocoena | 11/12/1990 | Sennen Cove Beach | South-west | F |  | 27.75 |  |
| SW1991/14 | Phocoena phocoena | 18/01/1991 | Wherry Town Beach, Penzance | South-west | F | 189 | 138.75 |  |
| SW1991/17a | Phocoena phocoena | 05/02/1991 | Hope Beach, Shanklin | Channel | F | 137 | 37.70 |  |
| SW1991/20 | Phocoena phocoena | 07/02/1991 | Southwold | East coast | F | 132 | 14.83 |  |
| SW1991/19b | Phocoena phocoena | 12/02/1991 | Porthmeor, (nr St. Ives) | South-west | F | 131 | 16.07 |  |
| SW1991/28 | Phocoena phocoena | 07/03/1991 | Hornsea | East coast | F | 132 | 7.93 |  |
| SW1991/29 | Phocoena phocoena | 11/03/1991 | Bridlington | East coast | F | 110 | 11.93 |  |
| SW1991/36 | Phocoena phocoena | 02/04/1991 | Fort Victoria | Channel | F | 152 | 12.90 |  |
| SW1991/43 | Phocoena phocoena | 20/04/1991 | Filey Brigg, N.end of Filey Bay (nr Scarborough) | East coast | F | 148 | 4.76 |  |
| SW1991/48 | Phocoena phocoena | 25/04/1991 | Theddlethorpe, (nr Mablethorpe) | East coast | F | 120 | 9.88 |  |
| SW1991/54 | Phocoena phocoena | 13/05/1991 | Foulness Point | East coast | F | 128 | 19.67 |  |
| SW1991/59 | Phocoena phocoena | 07/06/1991 | Gorleston Beach | East coast | F | 82 | 7.66 |  |
| SW1991/61 | Phocoena phocoena | 10/06/1991 | Ferryside | West coast | F | 72 | 5.87 |  |
| SW1991/63 | Phocoena phocoena | 12/06/1991 | Skegness | East coast | F | 76 | 11.41 |  |
| SW1991/95 | Phocoena phocoena | 30/07/1991 | Great Yarmouth | East coast | F | 140 | 20.36 |  |
| SW1991/111 | Phocoena phocoena | 05/09/1991 | Boggle Hole, S of Robin Hood's Bay | East coast | F | 134 | 36.98 |  |
| SW1991/116 | Phocoena phocoena | 16/09/1991 | Clacton-on-Sea | East coast | F | 152 | 32.56 |  |
| SW1991/120 | Phocoena phocoena | 23/09/1991 | Pembrey County Park | West coast | F | 117 | 86.99 |  |
| SW1991/135 | Phocoena phocoena | 21/10/1991 | Blackpool | West coast | F | 87 | 24.79 |  |
| SW1991/142 | Phocoena phocoena | 09/11/1991 | Towyn | West coast | F | 118 | 9.11 |  |
| SW1992/6 | Phocoena phocoena | 15/01/1992 | Sutton-on-Sea, (nr Mablethorpe) | East coast | F | 135 | 12.16 |  |
| SW1992/7 | Phocoena phocoena | 16/01/1992 | Borth | West coast | F | 131 | 18.64 |  |
| SW1992/202 | Phocoena phocoena | 13/10/1992 | Whitburn | East coast | F | 133 | 8.47 | 1.7381 |
| SW1992/215 | Phocoena phocoena | 07/12/1992 | Marazion | South-west | F | 190 | 3.13 |  |
| SW1993/10a | Phocoena phocoena | 18/01/1993 | nr Calgary, Mull | Strathclyde | F | 154 | 4.48 | 1.9318 |
| SW1993/10b | Phocoena phocoena | 23/01/1993 | South of Toab, Bay of Querndale, Mainland | Shetland | F | 111 | 21.58 | 10.2381 |
| SW1993/27 | Phocoena phocoena | 22/02/1993 | Seymour Tower | Channel | F | 182 | 78.29 |  |
| SW1993/41 | Phocoena phocoena | 16/03/1993 | Bodorgan Head, Anglesey | West coast | F | 144 | 35.54 |  |
| SW1993/63 | Phocoena phocoena | 10/05/1993 | Snettisham | East coast | F | 172 | 26.76 |  |
| SW1993/82a | Phocoena phocoena | 20/07/1993 | off Troup Head | Grampian | F | 156 | 1.88 | 0.8293 |
| SW1993/122 | Phocoena phocoena | 16/10/1993 | Tresaith beach | West coast | F | 156 | 5.12 |  |
| SW1993/124 | Phocoena phocoena | 22/10/1993 | Aberporth | West coast | F |  | 13.66 |  |
| SW1993/131 | Phocoena phocoena | 18/11/1993 | Saundersfoot | West coast | F | 100 | 14.65 |  |
| SW1993/133a | Phocoena phocoena | 24/11/1993 | Felixstowe | East coast | F | 168 | 24.56 |  |
| SW1994/7a | Phocoena phocoena | 23/01/1994 | Penbryn | West coast | F | 160 | 15.16 |  |
| SW1994/14a | Phocoena phocoena | 05/02/1994 | Saltburn (nr Invergordon) | Highland | F | 120 | 1.62 | 0.6915 |
| SW1994/20b | Phocoena phocoena | 18/02/1994 | Gosford Bay | Lothian | F | 112 | 24.43 | 8.1319 |
| SW1994/53 | Phocoena phocoena | 10/04/1994 | Morfa Dyffryn beach | West coast | F | 167 | 2.47 |  |
| SW1994/63 | Phocoena phocoena | 24/04/1994 | Chantry Point, Orford | East coast | F | 127 | 12.04 | 3.0682 |
| SW1994/68 | Phocoena phocoena | 28/04/1994 | Gunfleet Sands, 6 miles off Walton pier | East coast | F | 112 | 3.62 |  |
| SW1994/114 | Phocoena phocoena | 02/07/1994 | Briton Ferry | West coast | F | 82 | 62.26 |  |
| SW1994/115 | Phocoena phocoena | 03/07/1994 | Ynyslas | West coast | F | 77 | 16.73 |  |
| SW1994/120 | Phocoena phocoena | 12/07/1994 | RAF Pembrey bombing range | West coast | F | 108 | 24.93 |  |
| SW1994/143 | Phocoena phocoena | 24/08/1994 | Fairbourne (nr Barmouth) | West coast | F | 87 | 7.28 |  |
| SW1994/159b | Phocoena phocoena | 17/09/1994 | Balintore | Highland | F | 150 | 2.52 | 1.0737 |
| SW1994/185 | Phocoena phocoena | 30/12/1994 | Bosham harbour (nr Chichester) | Channel | F | 145 | 18.21 |  |
| SW1995/6a | Phocoena phocoena | 23/01/1995 | Longniddry | Lothian | F | 100 | 12.22 | 3.8046 |
| SW1995/34b | Phocoena phocoena | 13/03/1995 | Boddam, Mainland | Shetland | F | 147 | 2.90 | 1.4270 |
| SW1995/41a | Phocoena phocoena | 21/03/1995 | Echnaloch Bay, Burray | Orkney | F | 163 | 1.32 | 0.5876 |
| SW1995/61 | Phocoena phocoena | 07/06/1995 | Mablethorpe beach | East coast | F | 148 | 21.40 |  |
| SW1995/84 | Phocoena phocoena | 04/07/1995 | Penbryn | West coast | F | 154 | 3.12 |  |
| SW1995/85 | Phocoena phocoena | 05/07/1995 | Gorleston | East coast | F | 86 | 35.35 |  |
| SW1995/86 | Phocoena phocoena | 07/07/1995 | Trearddur Bay, Anglesey | West coast | F | 172 | 8.82 |  |
| SW1995/94 | Phocoena phocoena | 31/07/1995 | St Mary's Island lighthouse | East coast | F | 112 | 0.48 |  |
| SW1995/102 | Phocoena phocoena | 10/08/1995 | River Steeping near Gibraltar Point | East coast | F | 90 | 159.68 |  |
| SW1995/120a | Phocoena phocoena | 04/10/1995 | Snettisham | East coast | F | 107 | 10.44 |  |
| SW1995/122a | Phocoena phocoena | 06/10/1995 | Shandwick, Balintore | Highland | F | 104 | 2.69 | 1.6064 |
| SW1995/126 | Phocoena phocoena | 13/10/1995 | Withernsea | East coast | F | 95 | 44.15 |  |
| SW1995/142c | Phocoena phocoena | 22/12/1995 | 1.5 miles N of Rosemarkie | Highland | F | 139 | 4.44 | 2.1860 |
| SW1996/52a | Phocoena phocoena | 15/03/1996 | 2 miles S of Shandwick | Highland | F | 119 | 2.53 | 1.4348 |
| SW1996/69b | Phocoena phocoena | 31/03/1996 | W beach, nr Lossiemouth | Grampian | F | 126 | 5.54 | 2.7872 |
| SW1996/101d | Phocoena phocoena | 13/06/1996 | Arrochar | Strathclyde | F | 106 | 40.59 | 13.2184 |
| SW1996/126 | Phocoena phocoena | 25/07/1996 | off Whitby | East coast | F | 100 | 4.74 | 2.1512 |
| SW1996/139 | Phocoena phocoena | 27/08/1996 | 33 miles east of | At sea | F | 132 | 4.75 | 2.4565 |
| SW1996/150 | Phocoena phocoena | 22/09/1996 | Sheringham | East coast | F | 124 | 42.04 | 11.7241 |
| SW1996/160 | Phocoena phocoena | 23/10/1996 | Fishguard | West coast | F | 191 | 2.35 | 0.4938 |
| SW1996/175 | Phocoena phocoena | 09/12/1996 | Nolton Haven | West coast | F | 137 | 6.97 | 1.8256 |
| SW1997/21a | Phocoena phocoena | 22/01/1997 | North of Ramsey | Isle of Man | F | 119 | 33.46 | 8.1341 |
| SW1997/36 | Phocoena phocoena | 29/01/1997 | Bridlington Bay | East coast | F | 154 | 11.84 | 3.4000 |
| SW1997/72 | Phocoena phocoena | 18/04/1997 | off Cromer Point (nr Scarborough) | East coast | F | 110 | 2.75 | 1.2500 |
| SW1997/80 | Phocoena phocoena | 14/05/1997 | ca25.8 miles E of Runswick Bay | At sea | F | 123 | 3.71 | 1.5119 |
| SW1997/81 | Phocoena phocoena | 14/05/1997 | ca25.8 miles E of Runswick Bay | At sea | F | 129 | 7.18 | 2.2000 |
| SW1997/91 | Phocoena phocoena | 09/06/1997 | Black Pill, Swansea | West coast | F | 89 | 15.22 | 2.3333 |
| SW1997/93 | Phocoena phocoena | 13/06/1997 | Aberystwyth | West coast | F | 160 | 2.75 | 0.4176 |
| SW1997/94 | Phocoena phocoena | 19/06/1997 | Pembrey | West coast | F | 172 | 4.94 | 0.4011 |
| SW1997/96 | Phocoena phocoena | 23/06/1997 | Llanrhystud | West coast | F | 154 | 4.70 | 0.6933 |
| SW1997/97 | Phocoena phocoena | 24/06/1997 | Pendine Sands | West coast | F | 70 | 24.69 | 3.3898 |
| SW1997/102 | Phocoena phocoena | 03/07/1997 | Hunstanton | East coast | F | 84 | 16.55 | 6.5714 |
| SW1997/103 | Phocoena phocoena | 03/07/1997 | Thurlestone sands | South-west | F | 117 | 38.00 | 3.1447 |
| SW1997/111 | Phocoena phocoena | 10/07/1997 | BYCATCH 8km off Ulrome | East coast | F | 126 | 11.43 | 4.6778 |
| SW1997/113 | Phocoena phocoena | 13/07/1997 | Pembrey Country Park | West coast | F | 78 | 21.17 | 4.4400 |
| SW1997/118 | Phocoena phocoena | 18/07/1997 | Prestatyn | West coast | F | 172 | 5.29 | 0.9412 |
| SW1997/125b | Phocoena phocoena | 24/07/1997 | Unknown | Western Isles | F | 161 | 4.97 | 2.4205 |
| SW1997/135d | Phocoena phocoena | 20/08/1997 | Minches | Western Isles | F | 143 | 8.27 | 3.4444 |
| SW1997/137c | Phocoena phocoena | 26/08/1997 | Burray Ness | Orkney | F | 152 | 4.88 | 2.0690 |
| SW1997/138 | Phocoena phocoena | 07/09/1997 | Squires Gate (nr Blackpool) | West coast | F | 118 | 53.37 | 8.7500 |
| SW1997/142b | Phocoena phocoena | 11/09/1997 | 11¾ miles northeast of Kettleness, North Yorkshire | East coast | F | 132 | 6.64 |  |
| SW1997/152 | Phocoena phocoena | 07/10/1997 | Seasalter (nr Whitstable) | East coast | F | 107 | 59.20 | 12.7143 |
| SW1997/174 | Phocoena phocoena | 26/11/1997 | Gorleston | East coast | F | 147 | 15.37 | 4.3667 |
| SW1997/178 | Phocoena phocoena | 05/12/1997 | Whitley Bay beach | East coast | F | 156 | 5.00 | 0.8023 |
| SW1997/186.2 | Phocoena phocoena | 22/12/1997 | Whitley Sands | East coast | F | 145 | 1.47 |  |
| SW1998/2a | Phocoena phocoena | 06/01/1998 | Sand, Mainland | Shetland | F | 120 | 4.19 | 1.5889 |
| SW1998/4 | Phocoena phocoena | 09/01/1998 | North End, Mablethorpe | East coast | F | 100 | 11.05 | 4.5732 |
| SW1998/35b | Phocoena phocoena | 28/02/1998 | Black Rocks, nr Troon | Strathclyde | F | 161 | 15.83 | 5.9022 |
| SW1998/43a | Phocoena phocoena | 08/03/1998 | nr Portessie | Grampian | F | 152 | 1.96 | 0.8936 |
| SW1998/50 | Phocoena phocoena | 13/03/1998 | Westward Ho! beach | South-west | F | 131 | 11.90 | 1.5376 |
| SW1998/73a | Phocoena phocoena | 14/04/1998 | Echnaloch Bay, Burray | Orkney | F | 152 | 12.39 | 7.1264 |
| SW1998/75 | Phocoena phocoena | 20/04/1998 | 1 mile east of Cromer Point, north of Scarborough | East coast | F | 114 | 5.32 | 1.4747 |
| SW1998/97 | Phocoena phocoena | 21/05/1998 | Caldy Island | West coast | F | 133 | 20.56 | 3.1923 |
| SW1998/105b | Phocoena phocoena | 10/06/1998 | Findochty, Buckie | Grampian | F | 140 | 3.41 | 1.7742 |
| SW1998/129 | Phocoena phocoena | 07/07/1998 | Sea Palling | East coast | F | 154 | 18.37 | 5.5357 |
| SW1998/167a | Phocoena phocoena | 09/09/1998 | off Hurst, nr Lymington | Channel | F | 113 | 47.73 | 6.5676 |
| SW1998/187 | Phocoena phocoena | 03/11/1998 | Salthouse | East coast | F | 164 | 3.59 | 2.1294 |
| SW1998/191 | Phocoena phocoena | 09/11/1998 | Aberystwyth | West coast | F | 157 | 3.16 | 0.3581 |
| SW1998/198 | Phocoena phocoena | 27/11/1998 | Ynyslas | West coast | F | 123 | 6.22 | 0.9888 |
| SW1999/10 | Phocoena phocoena | 17/01/1999 | Druridge Bay | East coast | F | 148 | 1.52 | 0.3789 |
| SW1999/10a | Phocoena phocoena | 18/01/1999 | Troon | Strathclyde | F | 160 | 4.09 | 1.0526 |
| SW1999/17 | Phocoena phocoena | 28/01/1999 | South Bay, Bridlington | East coast | F | 101 | 8.30 | 3.4211 |
| SW1999/30a | Phocoena phocoena | 17/02/1999 | 9-10 miles southwest of Ailsa Craig | Strathclyde | F | 140 | 8.97 | 2.5955 |
| SW1999/40 | Phocoena phocoena | 04/03/1999 | near Westminster Bridge | East coast | F | 143 | 4.17 | 0.5910 |
| SW1999/48 | Phocoena phocoena | 12/03/1999 | 2 miles east of Sizewell Power Station | East coast | F | 115 | 5.10 | 1.3820 |
| SW1999/54a | Phocoena phocoena | 19/03/1999 | River Ythan | Grampian | F | 127 | 4.72 | 2.1099 |
| SW1999/62b | Phocoena phocoena | 26/03/1999 | nr Sandsound, Mainland | Shetland | F | 148 | 3.54 | 1.4894 |
| SW1999/71 | Phocoena phocoena | 05/04/1999 | North Ferriby | East coast | F | 98 | 13.26 | 2.6316 |
| SW1999/72a | Phocoena phocoena | 07/04/1999 | Ardersier | Highland | F | 149 | 0.95 | 0.2782 |
| SW1999/72d | Phocoena phocoena | 09/04/1999 | Alturlie Point, nr Inverness | Highland | F | 107 | 7.97 | 3.0460 |
| SW1999/74 | Phocoena phocoena | 10/04/1999 | Church Lane, Theddlethorpe | East coast | F | 118 | 5.26 | 1.9222 |
| SW1999/76a | Phocoena phocoena | 12/04/1999 | Charlestown, nr Dunfermline | Fife | F | 107 | 2.09 | 0.9222 |
| SW1999/90a | Phocoena phocoena | 20/04/1999 | Slatrach Bay, Kerrera | Strathclyde | F | 114 | 10.74 | 4.6118 |
| SW1999/96 | Phocoena phocoena | 01/05/1999 | Wallog | West coast | F | 111 | 6.17 | 1.0436 |
| SW1999/148c | Phocoena phocoena | 01/08/1999 | Rubha Garbh, nr Barcaldine | Strathclyde | F | 155 | 1.45 | 0.7500 |
| SW1999/174b | Phocoena phocoena | 23/09/1999 | Moreton, Wirral | West coast | F | 114 | 9.15 | 2.4773 |
| SW1999/189 | Phocoena phocoena | 26/10/1999 | Penbryn | West coast | F | 116 | 9.62 | 1.3684 |
| SW1999/192 | Phocoena phocoena | 29/10/1999 | Barmouth | West coast | F | 148 | 12.15 | 1.7229 |
| SW1999/196a | Phocoena phocoena | 12/11/1999 | Dunnet Bay, Caithness | Highland | F | 147 | 3.45 | 1.3511 |
| SW1999/202 | Phocoena phocoena | 05/12/1999 | Pont LLyfni | West coast | F | 113 | 4.79 | 1.0000 |
| SW1999/208 | Phocoena phocoena | 26/12/1999 | Langland Bay, Gower | West coast | F | 125 | 10.73 | 0.6477 |
| SW2000/13 | Phocoena phocoena | 06/02/2000 | Marazion | South-west | F | 130 | 6.25 | 0.9011 |
| SW2000/33 | Phocoena phocoena | 29/02/2000 | River Blyth at Blyth | East coast | F | 109 | 25.01 | 7.2353 |
| SW2000/37 | Phocoena phocoena | 03/03/2000 | West Dale beach | West coast | F | 140 | 16.89 | 3.2208 |
| SW2000/50 | Phocoena phocoena | 14/03/2000 | East of Battersea Bridge (south bank of Thames) | East coast | F | 148 | 17.79 | 2.5732 |
| SW2000/53 | Phocoena phocoena | 15/03/2000 | Fishguard | West coast | F | 115 | 5.94 | 0.9140 |
| SW2000/55 | Phocoena phocoena | 19/03/2000 | Lowestoft | East coast | F | 134 | 10.94 | 1.7711 |
| SW2000/73 | Phocoena phocoena | 02/04/2000 | off Sizewell | East coast | F | 152 | 7.41 | 1.2619 |
| SW2000/74b | Phocoena phocoena | 03/04/2000 | Footdee, Aberdeen | Grampian | F | 122 | 3.64 | 1.4947 |
| SW2000/81 | Phocoena phocoena | 12/04/2000 | Between Waxham and Horsey | East coast | F | 150 | 14.52 | 4.3571 |
| SW2000/83a | Phocoena phocoena | 23/04/2000 | St. Cyrus Nature Reserve | Grampian | F | 106 | 4.74 | 1.8913 |
| SW2000/140a | Phocoena phocoena | 09/08/2000 | Blairmore | Strathclyde | F | 128 | 22.38 | 6.9101 |
| SW2000/164 | Phocoena phocoena | 08/10/2000 | North Sea off Bridlington | East coast | F | 122 | 16.14 | 5.2558 |
| SW2000/169 | Phocoena phocoena | 21/10/2000 | 20 miles SE of Bridlington | East coast | F | 132 | 10.63 | 3.3000 |
| SW2000/170 | Phocoena phocoena | 21/10/2000 | 20 miles SE of Bridlington | East coast | F | 152 | 1.44 | 0.3667 |
| SW2000/174 | Phocoena phocoena | 25/10/2000 | Black Rock Sands | West coast | F | 126 | 13.82 | 2.4674 |
| SW2001/15b | Phocoena phocoena | 18/01/2001 | Lunan Bay, Montrose | Tayside | F | 148 | 3.19 | 1.4396 |
| SW2001/21 | Phocoena phocoena | 24/01/2001 | Crow Point, near Braunton Burrows | South-west | F | 171 | 2.65 | 0.1678 |
| SW2001/21a | Phocoena phocoena | 24/01/2001 | Peterhead | Grampian | F | 117 | 8.02 | 2.7222 |
| SW2001/21b | Phocoena phocoena | 25/01/2001 | Dunoon | Strathclyde | F | 129 | 10.94 | 3.6170 |
| SW2001/25c | Phocoena phocoena | 28/01/2001 | Cambo Sands, Kingsbarns | Fife | F | 125 | 3.34 | 1.2935 |
| SW2001/30 | Phocoena phocoena | 07/02/2001 | south of Mill Rock, Woolacombe beach, Puttsborough | South-west | F | 129 | 5.05 | 0.4526 |
| SW2001/36 | Phocoena phocoena | 17/02/2001 | Gillingham | East coast | F | 114 | 13.64 | 2.8090 |
| SW2001/40 | Phocoena phocoena | 05/03/2001 | Swansea Beach | West coast | F | 105 | 12.46 | 1.2386 |
| SW2001/43a | Phocoena phocoena | 08/03/2001 | Carnoustie | Tayside | F | 114 | 1.93 | 0.7701 |
| SW2001/47 | Phocoena phocoena | 12/03/2001 | Thurlestone end of South Milton Sands | South-west | F | 168 | 5.15 | 0.4130 |
| SW2001/55a | Phocoena phocoena | 14/03/2001 | Braigh, Stornoway | Western Isles | F | 126 | 3.38 | 1.7416 |
| SW2001/83c | Phocoena phocoena | 19/04/2001 | Cullen golf course | Grampian | F | 121 | 18.23 | 8.0952 |
| SW2001/85 | Phocoena phocoena | 21/04/2001 | Aberarth, Aberaeron | West coast | F | 122 | 3.82 | 0.5591 |
| SW2001/92 | Phocoena phocoena | 30/04/2001 | 'The Lees', between Walton-on the-Naze and Frinton | East coast | F | 117 | 10.86 | 3.6000 |
| SW2001/107a | Phocoena phocoena | 17/05/2001 | Garvan, Lochaber | Highland | F | 110 | 5.88 |  |
| SW2001/127c | Phocoena phocoena | 22/06/2001 | Arrochar | Strathclyde | F | 110 | 12.84 | 4.3295 |
| SW2001/144 | Phocoena phocoena | 09/07/2001 | 8 miles south of Bridlington, off Bridlington | East coast | F | 82 | 6.09 | 1.7091 |
| SW2001/149 | Phocoena phocoena | 12/07/2001 | 6 miles south of Bridlington, in 2 metres of water | East coast | F | 138 | 12.94 | 3.5402 |
| SW2001/158 | Phocoena phocoena | 17/07/2001 | Pegwell Bay, Sandwich Bay | East coast | F | 119 | 13.82 | 2.0111 |
| SW2001/181b | Phocoena phocoena | 29/07/2001 | Creetown, Newton Stewart | Dumfries and Galloway | F | 164 | 61.44 | 13.1522 |
| SW2001/188 | Phocoena phocoena | 08/08/2001 | Heacham, Norfolk | East coast | F | 98 | 12.89 |  |
| SW2001/198 | Phocoena phocoena | 28/08/2001 | Cei Bach, Ceredigion | West coast | F | 82 | 8.79 |  |
| SW2001/206c | Phocoena phocoena | 14/09/2001 | Ronachan, Tarbert, Argyll | Strathclyde | F | 168 | 1.99 | 0.7701 |
| SW2001/206d | Phocoena phocoena | 15/09/2001 | Burghead | Grampian | F | 137 | 2.62 | 1.2727 |
| SW2001/208 | Phocoena phocoena | 16/09/2001 | the Leasowe end of Wallasey beach, Wirral | West coast | F | 120 | 31.41 | 8.4048 |
| SW2001/218 | Phocoena phocoena | 27/09/2001 | by-catch 5 miles south of Bridlington at 10m depth | East coast | F | 144 | 24.76 | 5.5055 |
| SW2001/229 | Phocoena phocoena | 06/10/2001 | Charmouth beach | Channel | F | 157 | 13.89 | 1.3444 |
| SW2001/251 | Phocoena phocoena | 09/11/2001 | Aberavon | West coast | F | 108 | 18.77 | 2.4607 |
| SW2001/259a | Phocoena phocoena | 22/11/2001 | Helensburgh | Strathclyde | F | 149 | 32.29 | 9.8144 |
| SW2001/282 | Phocoena phocoena | 29/12/2001 | Aberarth | West coast | F | 113 | 21.85 | 3.1304 |
| SW2002/3 | Phocoena phocoena | 04/01/2002 | Swansea | West coast | F | 122 | 7.02 | 0.6682 |
| SW2002/54 | Phocoena phocoena | 26/01/2002 | Heacham beach | East coast | F | 106 | 8.08 | 3.3218 |
| SW2002/95 | Phocoena phocoena | 07/02/2002 | Blackpool Central Beach, Blackpool | West coast | F | 156 | 8.64 | 1.7556 |
| SW2002/112a | Phocoena phocoena | 09/03/2002 | Levenwick Beach | Shetland | F | 97 | 10.08 |  |
| SW2002/179 | Phocoena phocoena | 28/04/2002 | Tan-y-Bwlch, Aberystwyth | West coast | F | 135 | 5.57 | 0.8804 |
| SW2002/187a | Phocoena phocoena | 09/05/2002 | St David'S Harbour | Fife | F | 119 | 14.79 |  |
| SW2002/187b | Phocoena phocoena | 11/05/2002 | Balmedie Country Park | Grampian | F | 108 | 6.18 |  |
| SW2002/199d | Phocoena phocoena | 01/06/2002 | Skeld, Mainland | Shetland | F | 155 | 1.42 | 0.5667 |
| SW2002/214 | Phocoena phocoena | 18/06/2002 | Tal-y-Bont | West coast | F | 133 | 6.55 | 1.3596 |
| SW2002/239a | Phocoena phocoena | 25/06/2002 | Newton Shore, Ayr | Strathclyde | F | 164 | 5.27 |  |
| SW2002/250 | Phocoena phocoena | 09/07/2002 | off Bridlington | East coast | F | 152 | 1.29 | 0.3551 |
| SW2002/262a | Phocoena phocoena | 15/07/2002 | off Bridlington | East coast | F | 117 | 8.70 | 2.5000 |
| SW2002/294e | Phocoena phocoena | 15/08/2002 | Kames Bay, Isle of Bute | Strathclyde | F | 120 | 20.53 | 4.5934 |
| SW2002/308 | Phocoena phocoena | 02/09/2002 | 5 miles east of Bridlington | East coast | F | 157 | 3.09 | 0.6516 |
| SW2002/311a | Phocoena phocoena | 06/09/2002 | Blairmore, Dunoon | Strathclyde | F | 150 | 36.78 | 9.1099 |
| SW2002/321c | Phocoena phocoena | 23/09/2002 | East Links, N. Berwick | Lothian | F | 160 | 2.67 | 0.7444 |
| SW2002/351c | Phocoena phocoena | 22/11/2002 | Otter Ferry, Loch Fyne | Strathclyde | F | 166 | 7.38 | 1.0610 |
| SW2002/361c | Phocoena phocoena | 04/12/2002 | Balemore, North Uist | Western Isles | F | 135 | 15.23 |  |
| SW2002/372b | Phocoena phocoena | 11/12/2002 | Pitenweem | Fife | F | 109 | 10.92 |  |
| SW2003/36g | Phocoena phocoena | 10/01/2003 | Balnakiel Beach | Highland | F | 133 | 2.81 |  |
| SW2003/112a | Phocoena phocoena | 23/02/2003 | Toward Point, Dunoon | Strathclyde | F | 109 | 2.05 |  |
| SW2003/104a | Phocoena phocoena | 19/02/2003 | near Port Ellen, Islay | Strathclyde | F | 136 | 8.31 | 2.3556 |
| SW2003/170a | Phocoena phocoena | 17/03/2003 | Collieston | Grampian | F | 104 | 24.59 |  |
| SW2003/190 | Phocoena phocoena | 02/04/2003 | Tywyn | West coast | F | 151 | 1.44 | 0.2069 |
| SW2003/192a | Phocoena phocoena | 03/04/2003 | Whitehills Harbour, Banff | Grampian | F | 123 | 3.30 | 1.6522 |
| SW2003/194 | Phocoena phocoena | 06/04/2003 | Minsmere Haven (nr the sluice) | East coast | F | 139 | 10.84 | 2.7143 |
| SW2003/198b | Phocoena phocoena | 11/04/2003 | Crovie | Grampian | F | 125 | 3.65 |  |
| SW2003/219a | Phocoena phocoena | 26/04/2003 | St Monance | Fife | F | 149 | 21.90 | 8.0465 |
| SW2003/221 | Phocoena phocoena | 27/04/2003 | Hell's Mouth, Lleyn Peninsula | West coast | F | 153 | 4.86 | 0.6319 |
| SW2003/260h | Phocoena phocoena | 13/06/2003 | Girvan | Strathclyde | F | 173 | 53.81 | 15.5814 |
| SW2003/260j | Phocoena phocoena | 14/06/2003 | Sannox beach, Arran | Strathclyde | F | 154 | 51.59 | 16.0000 |
| SW2003/271 | Phocoena phocoena | 25/06/2003 | off Bridlington | East coast | F | 158 | 1.96 | 0.5187 |
| SW2003/274 | Phocoena phocoena | 27/06/2003 | Tywyn | West coast | F | 146 | 1.47 | 0.1843 |
| SW2003/278a | Phocoena phocoena | 02/07/2003 | off Elie harbour | Fife | F | 150 | 3.76 | 1.2299 |
| SW2003/296 | Phocoena phocoena | 23/07/2003 | off Bridlington | East coast | F | 154 | 2.80 | 0.6149 |
| SW2003/312 | Phocoena phocoena | 05/08/2003 | off Bridlington | East coast | F | 161 | 33.13 | 10.0000 |
| SW2003/337 | Phocoena phocoena | 27/08/2003 | Fishguard | West coast | F | 155 | 9.46 | 1.0341 |
| SW2003/344a | Phocoena phocoena | 09/09/2003 | Chanonry Point, Fortrose | Highland | F | 98 | 3.77 | 1.8000 |
| SW2003/353 | Phocoena phocoena | 26/09/2003 | Morfa Dyffryn | West coast | F | 111 | 7.52 | 1.1250 |
| SW2003/376f | Phocoena phocoena | 09/11/2003 | Lunan Bay | Tayside | F | 145 | 6.29 | 2.1250 |
| SW2003/380 | Phocoena phocoena | 16/11/2003 | Llanfwrog beach, Holyhead, Anglesey | West coast | F | 161 | 42.16 | 3.0682 |
| SW2004/37a | Phocoena phocoena | 27/01/2004 | Turnberry Bay | Strathclyde | F | 170 | 15.14 | 3.7416 |
| SW2004/90c | Phocoena phocoena | 23/02/2004 | Ascog, Bute | Strathclyde | F | 161 | 4.44 | 0.7241 |
| SW2004/91a | Phocoena phocoena | 26/02/2004 | Redcar beach | East coast | F | 125 | 6.52 | 2.8602 |
| SW2004/100 | Phocoena phocoena | 15/03/2004 | Eastbourne (near the pier) | Channel | F | 107 | 4.03 | 1.8842 |
| SW2004/108 | Phocoena phocoena | 17/03/2004 | Talacre beach (nr Prestatyn), Point of Ayr | West coast | F | 133 | 12.61 | 1.6818 |
| SW2004/120 | Phocoena phocoena | 23/03/2004 | Poppit Sands | West coast | F | 131 | 5.30 | 1.2211 |
| SW2004/123c | Phocoena phocoena | 25/03/2004 | Skateraw Harbour (W of) | Lothian | F | 151 | 9.32 | 1.5057 |
| SW2004/144 | Phocoena phocoena | 15/04/2004 | New Brighton beach, Wirral peninsula | West coast | F | 110 | 17.10 | 4.9195 |
| SW2004/154d | Phocoena phocoena | 01/05/2004 | Ardbeg Point, Rothesay | Strathclyde | F | 147 | 17.53 | 5.6279 |
| SW2004/167g | Phocoena phocoena | 14/05/2004 | E of Nairn | Highland | F | 126 | 16.02 | 5.7882 |
| SW2004/173b | Phocoena phocoena | 01/06/2004 | Arrochar | Strathclyde | F | 128 | 13.54 | 3.8471 |
| SW2004/179 | Phocoena phocoena | 08/06/2004 | Swansea | West coast | F | 126 | 28.46 | 2.3034 |
| SW2004/222d | Phocoena phocoena | 14/07/2004 | Gardenstown harbour | Grampian | F | 122 | 3.51 | 1.2809 |
| SW2004/277 | Phocoena phocoena | 14/09/2004 | Black Rock Sands, Porthmadog | West coast | F | 123 | 19.23 | 2.8696 |
| SW2004/291 | Phocoena phocoena | 28/09/2004 | Doniford beach, Watchet | South-west | F | 112 | 5.54 | 0.8424 |
| SW2004/316 | Phocoena phocoena | 16/10/2004 | Poppit Sands | West coast | F | 160 | 8.06 | 1.1099 |
| SW2004/354e | Phocoena phocoena | 15/12/2004 | Lunan Bay, Montrose | Tayside | F | 167 | 1.62 | 0.4945 |
| SW2005/1d | Phocoena phocoena | 02/01/2005 | nr Portavadie, Cowal | Strathclyde | F | 118 | 13.35 |  |
| SW2005/28 | Phocoena phocoena | 06/02/2005 | Climping beach, Littlehampton | Channel | F | 119 | 28.88 |  |
| SW2005/28c | Phocoena phocoena | 08/02/2005 | Philorth, Fraserburgh | Grampian | F | 165 | 4.82 | 1.4000 |
| SW2005/32b | Phocoena phocoena | 15/02/2005 | Queensferry | Lothian | F | 117 | 14.37 |  |
| SW2005/34b | Phocoena phocoena | 19/02/2005 | Dunoon | Strathclyde | F | 106 | 19.54 |  |
| SW2005/47b | Phocoena phocoena | 28/02/2005 | off Oronsay, Mull | Highland | F | 93 | 6.30 |  |
| SW2005/56g | Phocoena phocoena | 11/03/2005 | Spurn Head (just inside the tip) by lighthouse | East coast | F | 160 | 2.34 |  |
| SW2005/62d | Phocoena phocoena | 17/03/2005 | Kilcreggan, Roseneath | Strathclyde | F | 160 | 24.12 | 9.5909 |
| SW2005/66 | Phocoena phocoena | 21/03/2005 | Winterton beach, Winterton-on-Sea | East coast | F | 121 | 6.14 |  |
| SW2005/71 | Phocoena phocoena | 24/03/2005 | Penrhyn, Colwyn Bay | West coast | F | 118 | 28.51 |  |
| SW2005/72d | Phocoena phocoena | 24/03/2005 | Ardbeg, Islay | Strathclyde | F | 152 | 2.84 | 0.4213 |
| SW2005/92a | Phocoena phocoena | 09/04/2005 | Chanonry Ness, Fortrose | Highland | F | 140 | 1.03 |  |
| SW2005/115c | Phocoena phocoena | 01/05/2005 | Shandwick Bay | Highland | F | 116 | 3.32 | 1.2778 |
| SW2005/118 | Phocoena phocoena | 08/05/2005 | Castell Bach | West coast | F | 104 | 5.37 |  |
| SW2005/123 | Phocoena phocoena | 12/05/2005 | Aberdaron, Lleyn peninsula | West coast | F | 153 | 37.86 |  |
| SW2005/125a | Phocoena phocoena | 17/05/2005 | Roseisle, Burghead | Grampian | F | 113 | 6.52 | 1.3626 |
| SW2005/142a | Phocoena phocoena | 04/06/2005 | off Peterhead (44 miles E of) | At sea | F | 144 | 1.92 | 0.4739 |
| SW2005/150 | Phocoena phocoena | 09/06/2005 | Pembrey | West coast | F | 143 | 12.12 |  |
| SW2005/154 | Phocoena phocoena | 13/06/2005 | Mwnt | West coast | F | 79 | 1.81 |  |
| SW2005/162 | Phocoena phocoena | 22/06/2005 | Barmouth | West coast | F | 156 | 11.02 |  |
| SW2005/184 | Phocoena phocoena | 09/07/2005 | Nefyn, Lleyn peninsula | West coast | F | 163 | 11.23 |  |
| SW2005/253b | Phocoena phocoena | 14/10/2005 | Kirkcolm, Wigton | Dumfries and Galloway | F | 156 | 20.54 |  |
| SW2005/266 | Phocoena phocoena | 31/10/2005 | Camber Sands, Rye Village | Channel | F | 107 | 21.26 |  |
| SW2005/271b | Phocoena phocoena | 11/11/2005 | The Scar, Kirkcolm | Dumfries and Galloway | F | 149 | 14.52 |  |
| SW2005/285 | Phocoena phocoena | 04/12/2005 | Fishguard | West coast | F | 131 | 15.64 |  |
| SW2006/10a | Phocoena phocoena | 13/01/2006 | Balmedie, Aberdeen | Grampian | F | 105 | 4.10 |  |
| SW2006/82 | Phocoena phocoena | 05/03/2006 | Ainsdale beach, Ainsdale-on-Sea | West coast | F | 164 | 28.71 |  |
| SW2006/112 | Phocoena phocoena | 09/04/2006 | Aberporth | West coast | F | 149 | 3.19 |  |
| SW2006/163 | Phocoena phocoena | 20/05/2006 | Amroth | West coast | F | 173 | 8.89 |  |
| SW2006/172 | Phocoena phocoena | 09/06/2006 | Aberporth | West coast | F | 156 | 3.35 |  |
| SW2006/262 | Phocoena phocoena | 09/10/2006 | Poole | Channel | F | 158 | 138.83 |  |
| SW2006/278a | Phocoena phocoena | 13/11/2006 | Seamill, West Kilbride | Strathclyde | F | 156 | 6.28 |  |
| SW2007/28 | Phocoena phocoena | 28/01/2007 | Trevone, nr Padstow | South-west | F | 155 | 5.35 |  |
| SW2007/59b | Phocoena phocoena | 26/02/2007 | West Sands, St Andrews | Fife | F | 115 | 2.31 |  |
| SW2007/60a | Phocoena phocoena | 27/02/2007 | Belhaven Bay | Lothian | F | 154 | 2.55 |  |
| SW2007/94b | Phocoena phocoena | 21/04/2007 | Wemyss Bay | Strathclyde | F | 161 | 8.40 |  |
| SW2007/109 | Phocoena phocoena | 18/05/2007 | Black Rock Sands, Porthmadog | West coast | F | 160 | 4.24 |  |
| SW2007/114 | Phocoena phocoena | 26/05/2007 | Aberdovey | West coast | F | 116 | 3.40 |  |
| SW2007/128 | Phocoena phocoena | 30/06/2007 | Borth | West coast | F | 153 | 1.75 |  |
| SW2007/138 | Phocoena phocoena | 10/07/2007 | Borth | West coast | F | 80 | 2.95 |  |
| SW2007/157 | Phocoena phocoena | 06/08/2007 | Lytham St Anne's | West coast | F | 110 | 32.34 |  |
| SW2007/172 | Phocoena phocoena | 28/08/2007 | Herne Bay | East coast | F | 149 | 8.66 |  |
| SW2007/181b | Phocoena phocoena | 09/09/2007 | New Quay | West coast | F | 88 | 16.38 |  |
| SW2007/188d | Phocoena phocoena | 17/09/2007 | Portessie, Buckie | Grampian | F | 123 | 2.74 |  |
| SW2007/195 | Phocoena phocoena | 28/09/2007 | Criccieth | West coast | F | 157 | 3.43 |  |
| SW2007/196 | Phocoena phocoena | 29/09/2007 | Cromer | East coast | F | 93 | 6.30 |  |
| SW2007/224 | Phocoena phocoena | 15/11/2007 | Pembrey | West coast | F | 114 | 7.34 |  |
| SW2008/16c | Phocoena phocoena | 03/02/2008 | Girvan | Strathclyde | F | 129 | 7.74 |  |
| SW2008/23 | Phocoena phocoena | 11/02/2008 | 'Red Water', Western Cleddau at Little Milford | West coast | F | 164 | 24.13 |  |
| SW2008/31a | Phocoena phocoena | 15/02/2008 | Burghead | Grampian | F | 109 | 9.20 |  |
| SW2008/37 | Phocoena phocoena | 28/02/2008 | at sea (Dulas Bay) | West coast | F | 116 | 5.43 |  |
| SW2008/38d | Phocoena phocoena | 01/03/2008 | Ayr (south beach) | Strathclyde | F | 160 | 8.69 |  |
| SW2008/48g | Phocoena phocoena | 26/03/2008 | Portknockie harbour | Grampian | F | 94 | 3.74 |  |
| SW2008/49 | Phocoena phocoena | 26/03/2008 | Bridlington (South Sands) | East coast | F | 117 | 10.38 |  |
| SW2008/52 | Phocoena phocoena | 30/03/2008 | Fraisthorpe | East coast | F | 164 | 2.51 |  |
| SW2008/59 | Phocoena phocoena | 07/04/2008 | Boyndie Bay, Banff | Grampian | F | 112 | 6.15 |  |
| SW2008/60 | Phocoena phocoena | 10/04/2008 | between Cleveleys and Blackpool | West coast | F | 158 | 1.67 |  |
| SW2008/73 | Phocoena phocoena | 27/04/2008 | Tywyn | West coast | F | 114 | 8.45 |  |
| SW2008/84 | Phocoena phocoena | 19/05/2008 | Tankerton, Whitstable | East coast | F | 125 | 15.84 |  |
| SW2008/123b | Phocoena phocoena | 18/07/2008 | Aberavon | West coast | F | 119 | 18.04 |  |
| SW2009/2 | Phocoena phocoena | 07/01/2009 | Brixham, St Mary's Bay | South-west | F | 145 | 11.14 |  |
| SW2009/69 | Phocoena phocoena | 04/03/2009 | Menie Links, Balmedie, Aberdeen. | Grampian | F | 155 | 1.95 |  |
| SW2009/82 | Phocoena phocoena | 16/03/2009 | Menie Links, Balmedie, Aberdeen, Grampian | Grampian | F | 125 | 18.25 |  |
| SW2009/110 | Phocoena phocoena | 13/04/2009 | Oxwich, Gower, Swansea. | West coast | F | 162 | 16.02 |  |
| SW2009/198 | Phocoena phocoena | 09/06/2009 | East Beach, Nairn, Highland | Highland | F | 154 | 1.80 |  |
| SW2009/256 | Phocoena phocoena | 31/07/2009 | Dunnet Bay, Thurso, Highland | Highland | F | 160 | 0.66 |  |
| SW2009/422 | Phocoena phocoena | 16/12/2009 | Whiting Bay, Isle of Arran | Strathclyde | F | 165 | 4.58 |  |
| SW2010/1 | Phocoena phocoena | 02/01/2010 | Bridlington | East coast | F | 157 | 0.69 |  |
| SW2010/42 | Phocoena phocoena | 11/02/2010 | Cart Gap beach, Eccles on Sea | East coast | F | 162 | 4.11 |  |
| SW2010/51 | Phocoena phocoena | 03/02/2010 | West Sands, St Andrews | Fife | F | 122 | 4.74 |  |
| SW2010/64 | Phocoena phocoena | 27/02/2010 | Blyth beach | East coast | F | 120 | 2.35 |  |
| SW2010/220 | Phocoena phocoena | 13/07/2010 | Sandbank, Dunoon, Argyll & Bute | Strathclyde | F | 88 | 11.82 |  |
| SW2010/228 | Phocoena phocoena | 29/07/2010 | Ynyslas | West coast | F | 153 | 2.82 |  |
| SW2010/272 | Phocoena phocoena | 30/08/2010 | New Quay | West coast | F | 145 | 10.98 |  |
| SW2010/315 | Phocoena phocoena | 28/09/2010 | Skinningrove beach | East coast | F | 145 | 0.74 |  |
| SW2010/342 | Phocoena phocoena | 19/10/2010 | Sennen Cove | South-west | F | 147 | 4.95 |  |
| SW2010/365 | Phocoena phocoena | 05/11/2010 | Barassie beach, Troon | Strathclyde | F | 156 | 6.08 |  |
| SW2011/16 | Phocoena phocoena | 02/01/2011 | Port Bannatyne, Rothesay, Isle of Bute | Strathclyde | F | 160 | 11.61 |  |
| SW2011/136 | Phocoena phocoena | 20/03/2011 | Head of Loch Sunart, Strontian | Highland | F | 162 | 4.05 |  |
| SW2011/169 | Phocoena phocoena | 29/04/2011 | Tankerton Bay | East coast | F | 110 | 9.71 |  |
| SW2012/19 | Phocoena phocoena | 17/01/2012 | Wiseman's Bridge | West coast | F | 152 | 10.50 |  |
| SW2012/105 | Phocoena phocoena | 09/03/2012 | Shell Bay, Elie | Fife | F | 119 | 2.64 |  |
| SW2012/114 | Phocoena phocoena | 20/03/2012 | Brighton Beach | Channel | F | 158 | 50.46 |  |
| SW2012/137 | Phocoena phocoena | 05/04/2012 | Ramsey Sound | West coast | F | 126 | 21.62 |  |
| SW2012/185 | Phocoena phocoena | 16/04/2012 | Findhorn, Moray | Grampian | F | 104 | 3.69 |  |
| SW2012/256 | Phocoena phocoena | 22/06/2012 | Rhosilli | West coast | F | 76 | 6.80 |  |
| SW2012/313 | Phocoena phocoena | 21/07/2012 | Lossiemouth Beach East | Grampian | F | 154 | 2.38 |  |
| SW2012/358 | Phocoena phocoena | 06/08/2012 | Brook beach | Channel | F | 111 | 14.52 |  |
| SW2012/369 | Phocoena phocoena | 05/09/2012 | Paignton | South-west | F | 178 | 20.94 |  |
